# Prevalence of Japanese encephalitis in pigs in Mainland China during 2000–2024: a systemic review and meta-analysis

**DOI:** 10.3389/fvets.2025.1534114

**Published:** 2025-02-07

**Authors:** Xue-Tong Liu, Li-Dong Jiang, Yu-Ting Lin, Ran Zhao, Qi Wang, Shu-Ying Zhang, Emad Beshir Ata, Xin Liu, Yuan Wang, Zi-Xuan Liu, Cui Xu, Ying Xiao, Yi-Fan Wang, Xue Leng, Qing-Long Gong, Rui Du

**Affiliations:** ^1^College of Veterinary Medicine, Jilin Agricultural University, Changchun, China; ^2^Ginseng and Antler Products Testing Center of the Ministry of Agricultural PRC, Jilin Agricultural University, Changchun, China; ^3^College of Chinese Medicine Materials, Jilin Agricultural University, Changchun, China; ^4^Department of Parasitology and Animal Diseases, Veterinary Research Institute, National Research Centre, Giza, Egypt; ^5^Department of Veterinary Medicine, College of Agriculture, Yanbian University, Yanji, China

**Keywords:** Japanese encephalitis, prevalence, pigs, zoonosis, meta-analysis

## Abstract

**Background:**

Japanese encephalitis (JE) is an acute viral disease transmitted mainly by mosquitoes, primarily affecting Southeast Asia, and the Western Pacific. This study aimed to analyze the factors contributing to JE occurrence in pigs across China.

**Methods:**

A systematic search was done using six databases for the published epidemiological studies on porcine JE, including the Chinese Web of Knowledge (CNKI), Wan Fang Database, ScienceDirect, Web of Science, VIP Chinese Journal Database, and PubMed.

**Results:**

A meta-analysis of 31 studies from 2000 to 2024 found an overall prevalence of 35.2% (95% CI: 25.1–46.1). The highest prevalence occurred between 2010 and 2015 at 53.4% (95% CI: 44.2–80.6), from 2010 to 2015, increased precipitation and favorable annual temperatures led to the proliferation of mosquitoes, causing Japanese Encephalitis outbreaks among swine. While the lowest was 2.5% (95% CI: 0.2–6.6) in temperate climates. Serum samples showed the highest prevalence 38.1% (95% CI: 27.9–48.9), and ELISA testing had a higher detection rate 38.2% (95% CI: 24.5–52.9). In the farming mode subgroup, the highest prevalence was observed in the large-scale farming mode at 40.9% (95% CI: 26.4–66.3).

**Conclusion:**

The study highlights the spread of JE across China and suggests that it may be underrecognized in some areas. Continuous monitoring and improvements in farming practices are essential for controlling the disease.

## 1 Introduction

The farm animals play an essential role in maintaining the global food security (1, 2). They were subjected to different pathogens that affected their productivity (3–5), especially the swine sector is affected by different pathogens (6–9).

Japanese encephalitis (JE) also known as Epidemic encephalitis B (10), is a naturally occurring epidemic caused by the insect-born *Japanese encephalitis virus (JEV)*; a member of the flavivirus group (11, 12) which leads to neurological disorders by affecting the central nervous system of animals (13, 14), and has been classified as a category II of animal diseases in China (15). Because of the disease zoonotic potentiality, the World Health Organization (WHO) recommends human immunization as the most effective means to control the JE (16). Though the disease can occur year-round (17), it shows distinct seasonality, peaking in summer and fall (18). Outbreaks can also be triggered by poor feeding management, unsanitary conditions, and abnormal climate changes (19). The *JEV* is transmitted by mosquito vectors (20), with birds and bats serving as the primary reservoir hosts. It has a broad host range, including various animal species and humans. Notably, pigs, horses, and humans exhibit observable clinical symptoms, while other infected animals generally do not show significant signs of infection (21). The pigs play a crucial role mainly as amplification hosts during human outbreaks (17, 22, 23). Pigs may exhibit prolonged viremia, lasting from weeks to months, and are susceptible to the disease at any age (24). Infection of sows during gestation period might result in abortion, stillbirth, or give birth to mummified fetuses. While, in boars, infection causes swollen testes, reduced sperm quality, diminished libido, and eventual reproductive failure (25). The main route of infection is through biting of mosquitoes vector; mainly the Culex tritaeniorhynchus, fed on diseased pigs. The virus can survive and replicate within mosquitoes, which then transmit it to other pigs and people through bites (26). Pigs play a crucial role as amplifying hosts in the JE transmission cycle, alongside water birds (27). They can develop viremia sufficient to sustain transmission and are frequently linked to epizootic spillover leading to human JE cases (27). Recent studies have revealed that pigs can shed *JEV* through multiple routes and maintain persistent infections, suggesting a potential for vector-free transmission among pigs (27, 28). Pigs are primary reservoirs for the *JEV*, which mosquitoes can transmit to humans. In Mainland China, with the improvement of living standards, the number of pigs is increasing gradually. According to government statistics, in 2014, the number of pigs in Mainland China was estimated as approximately 465,827,000, and pork is commonly consumed by the Chinese population (29). Therefore, pigs are the most important potential source for Japanese encephalitis infection in humans. Surprisingly, the virus can overcome the vector mosquito route and spread between swine herds through highly contagious oro-nasal secretions (30). The virus persists even during winter when mosquito populations are low (31), which complicate the eradication efforts. Consequently, the disease poses a serious threat to the pig farming industry, causing significant economic losses and hindering industry growth in China and globally (32).

The epidemiological situation of the disease varies between the countries but mainly found across East and Southeast Asia, including China, Japan, Korea, India, Thailand, and Vietnam (33). The causative agent can infect multiple host species including equine and swine. The *JEV* P3 strain was first isolated in China in 1949 and remained endemic for the next 60 years (34). Mosquito species are the primary vectors of this virus, while pigs are the main reservoirs that promote the transmission of *JEV* from animals to humans (26, 35). However, China has a vast hog farming industry. According to statistics, the number of pigs farrowed reached 735.1 million in 2014 (36). In 2015, 624 human cases of JE were reported in China, 19 of which were fatal (26). Furthermore, the *JEV* has become a major pathogen causing reproductive disorders in pigs, leading to severe economic losses (32), making it also a potential threat to human health (24).

To our knowledge, no comprehensive systematic analysis of the overall prevalence of this disease has been conducted in China. Thus, this systematic review and meta-analysis aimed to examine the prevalence of JE in Chinese swine herds and assess potential risk factors: including time of sampling, area of sample collection, testing method, and type of samples, in addition to the evaluation of raw data from the included studies, geographic factors such as longitude, latitude, elevation, rainfall, humidity, temperature, and climate conditions were examined to determine their relationship to the prevalence of the disease.

## 2 Materials and methods

### 2.1 Search strategy

This study followed the PRISMA guidelines (Supplementary Table S1) (37, 38). Literature related to porcine JE was retrieved from six databases, including PubMed, ScienceDirect, Web of Science, CNKI, Wan Fang Data Knowledge Service Platform, and Wipro Chinese Journal Database. We reviewed all national literature on porcine JE published between January 1, 2000, and May 8, 2024, with sampling dates from 1997 to 2021.

The following formulas and MeSH terms were used in PubMed “Swine,” “Pig,” “Encephalitis, Japanese” and “China” were used in PubMed. Boolean operators “AND” were used to connect MeSH terms and “OR” to connect the entry terms.

In ScienceDirect, we searched for “Prevalence,” “Swine,” “Japanese B Encephalitis,” and “China.” In Web of Science, “Japanese B Encephalitis,” “Swine,” and “Prevalence” were used as keywords. In three Chinese databases, “liuxingxingyixingnaoyan (in Chinese)” and “zhu (in Chinese)” or “yixingnaoyan (in Chinese)” and “zhu (in Chinese)” were used to search with fuzzy search and synonym expansion in advanced searches. Detailed search formulas were provided in Supplementary Table S2. Retrieved articles were sorted and screened with Endnote X21 (version 21.2.0.17387).

Studies were included if they met the following criteria: (1) Study subjects must be pigs; (2) The objective must be to assess the prevalence of JE infection; (3) Data must include the total number of pigs tested and those testing positive; (4) The study must be conducted in China; (5) The study design must be cross-sectional; (6) The study must be published in Chinese or English. (7) The pigs must be naturally infected. Studies not meeting these criteria were excluded. Duplicate studies and review articles (non-research papers) were also excluded.

### 2.2 Data extraction and quality assessment

Four reviewers utilized a standardized data collection form to extract data for the meta-analysis (39). Discrepancies between reviewers or uncertainties regarding study quality were resolved by the lead author. The extracted data included: first author, sampling year, publication year, sample type, geographic area, province, latitude and longitude, elevation, mean annual temperature, humidity, max/min temperature, max daily precipitation, climate, testing method, age, sex, season of collection, feeding method, mode of swine husbandry, total swine samples, and number of positive samples for JE.

The quality of the publications was assessed using a standardized scoring method (40). Each study was evaluated on specific criteria (such as randomized sampling, assay clarity, detailed sampling methods, clear sampling timeframes, and inclusion of four or more relevant factors). Each study received a score from 0 to 5 on a standardized scale.

### 2.3 Data analysis

All calculations, including those related to the prevalence of porcine JE, were conducted using R software (version 4.0.2) using data from multiple studies. The double-arcsine transform (PFT) were selected for rate conversion based on these results and prior research findings (Table 1) (41).

**Table 1:** Normal distribution test for the normal rate and the different conversion of the normal rate.

Conversion form	*W*	*P*
PRAW	0.942	0.093
PLN	0.901	0.008
PLOGIT	0.979	0.793
PAS	0.966	0.406
PFT	0.969	0.500

“PRAW”: original rate; “PLN”: logarithmic conversion; “PLOGIT”: logit transformation; “PAS”: arcsine transformation; “PFT”: double-arcsine transformation; “NaN”: meaningless number; “NA”: missing data.

The PFT formula is:

$$\begin{array}{l} t=\arcsin\left\{\mathrm{sqrt}\left[\begin{array}{l} r/\\ \left(n+1\right) \end{array}\right]\right\}+\arcsin\left\{\mathrm{sqrt}\left[\begin{array}{l} \left(r+1\right)/\\ \left(n+1\right) \end{array}\right]\right\}\;se\left(t\right)\\ =\mathrm{sqrt}\left[\begin{array}{l} 1/\\ \left(n+0.5\right) \end{array}\right] \end{array}$$

$$p={\left[\sin\left(t/2\right)\right]}^{2}$$

Note: t: conversion prevalence; r = positive rate; n = sample size; se = standard deviation.

Forest plots were employed to visualize the results and assess heterogeneity between studies. Heterogeneity was calculated using Cochran’s Q-test and the *I*^2^ statistic, with 50% as the critical value for *I*^2^. The *χ*^2^ test (*p* < 0.05) was also applied. *I*^2^ < 50%indicates low heterogeneity, suggesting that the differences in study results were primarily due to random errors. *I*^2^ ≥ 50% indicated high heterogeneity and significant inconsistency between study results, suggesting that other factors may contribute to the observed variations. In such cases, potential factors contributing to heterogeneity require further investigation. These methods were applied to assess the statistical significance of heterogeneity in the selected studies. When heterogeneity was evident, a random-effects model was employed for meta-analysis (42). Publication bias was evaluated with funnel plots, the trim-and-fill method, and Egger’s test. Studies suggested that different subgroups may produce varying funnel plots due to changes in prevalence over time (36). Thus, each subgroup was further evaluated through funnel plots and forest plots. Sensitivity analyses were conducted to determine if any single study significantly impacted the overall estimates (43).

Heterogeneity is a critical metric in meta-analyses; thus, accurate assessing is essential to identifying key factors for preventing JE infection in pigs nationwide. To explore potential sources of heterogeneity, subgroup analyses and univariate regression were employed to identify its predictors. The factors assessed included; geographic region (Northeast vs. other regions), sampling period (post–2015 vs. pre–2010 and 2010–2015), assay method (PCR vs. ELISA, RT-RAA, LAT), season (autumn vs. spring, summer, winter), sex (boars vs. sows), age classification (nursery pigs vs. Weaned piglets and fattening pigs), sample type (serum vs. organization, brain tissue, blood), feeding system (large-scale vs. free-range), and study quality (high-quality vs. medium-quality studies). To further explore other potential sources of heterogeneity, we further assessed their geographic factors, in groups, which included longitude, latitude, elevation, rainfall, humidity, and climate.

This meta-analysis adhered to the PRISMA guidelines (Supplementary Table S1) (37, 38, 44). Correlations were analyzed for each subgroup based on testing method and region to identify heterogeneity sources. Heterogeneity in covariates was quantified using the R^2^ statistic. This meta-analysis lacked a review protocol and was not registered with the Cochrane Database. The R codes for this meta-analysis are available in Supplementary Table S3.

## 3 Results

**Figure 1:**
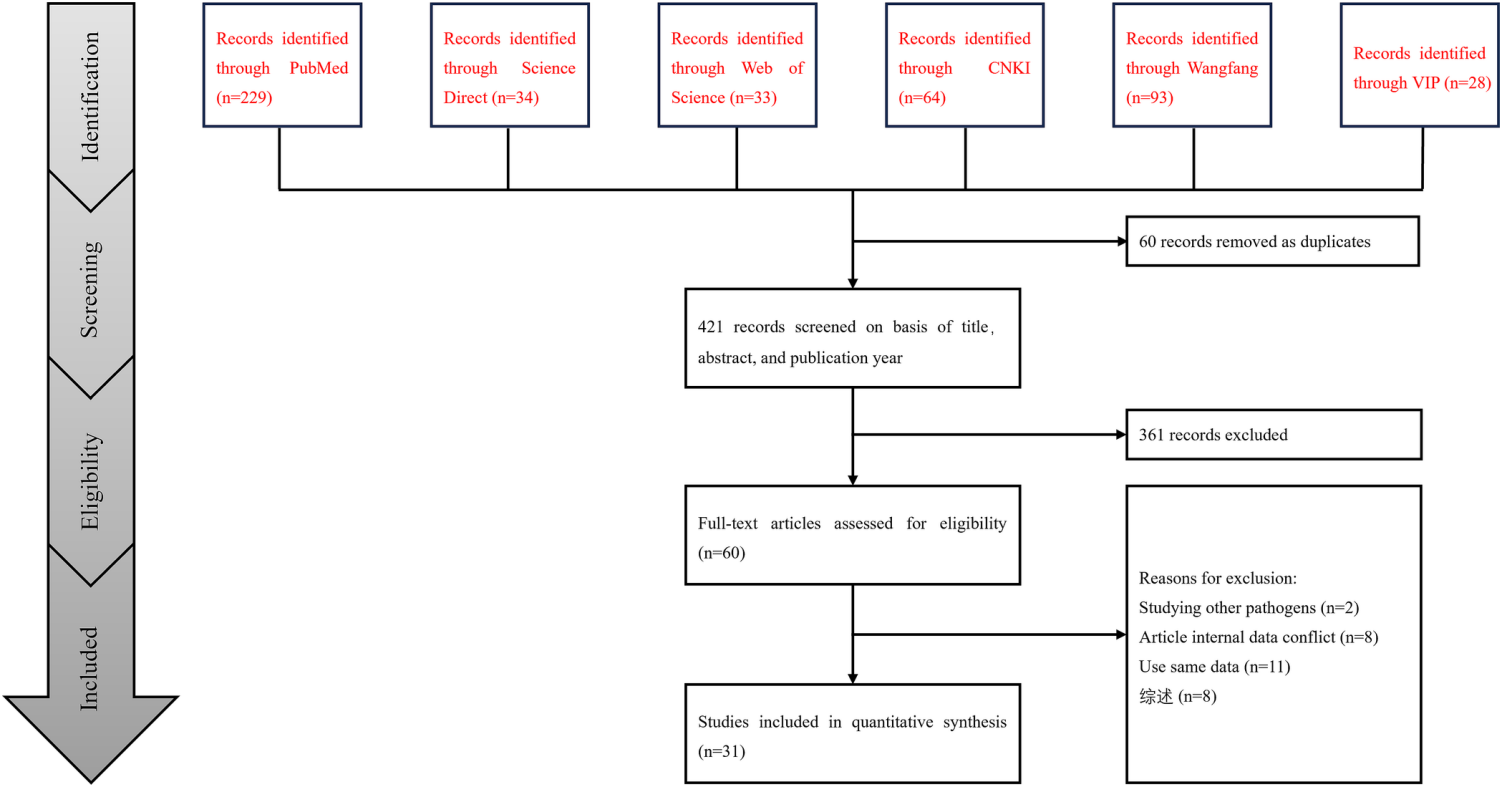
Flow diagram of eligible studies for searching and selecting.

A total of 481 studies were identified from six databases. A meta-analysis was performed on 31 studies that met the inclusion and exclusion criteria (Figure 1). Among the included studies, five had quality scores between 4 and 5, 26 scored between 2 and 3, and none scored between 0 and 1.

### 3.1 Publishing biased results

**Figure 2:**
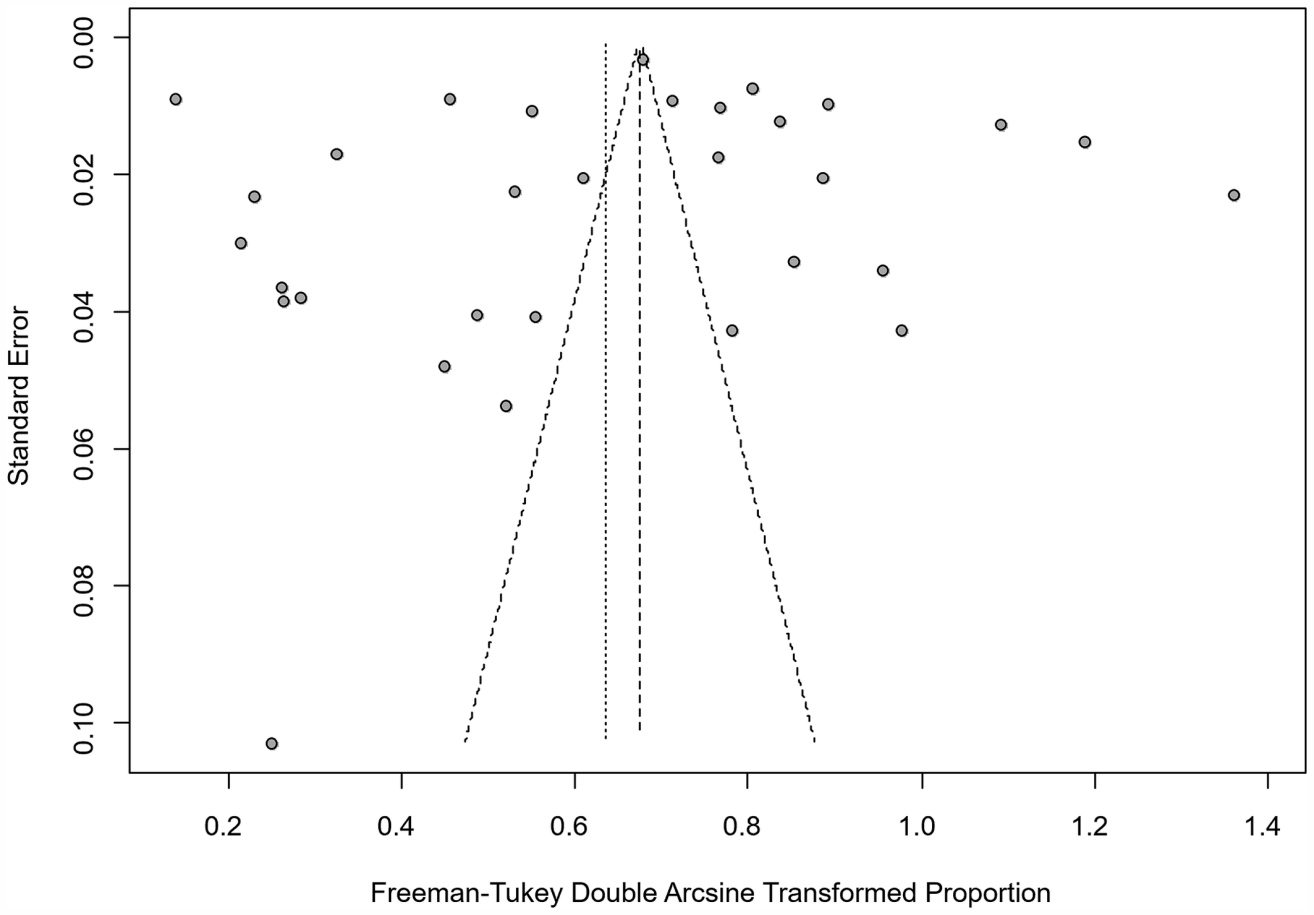
Funnel plot with pseudo 95% confidence interval limits for the examination of publication bias.

**Figure 3:**
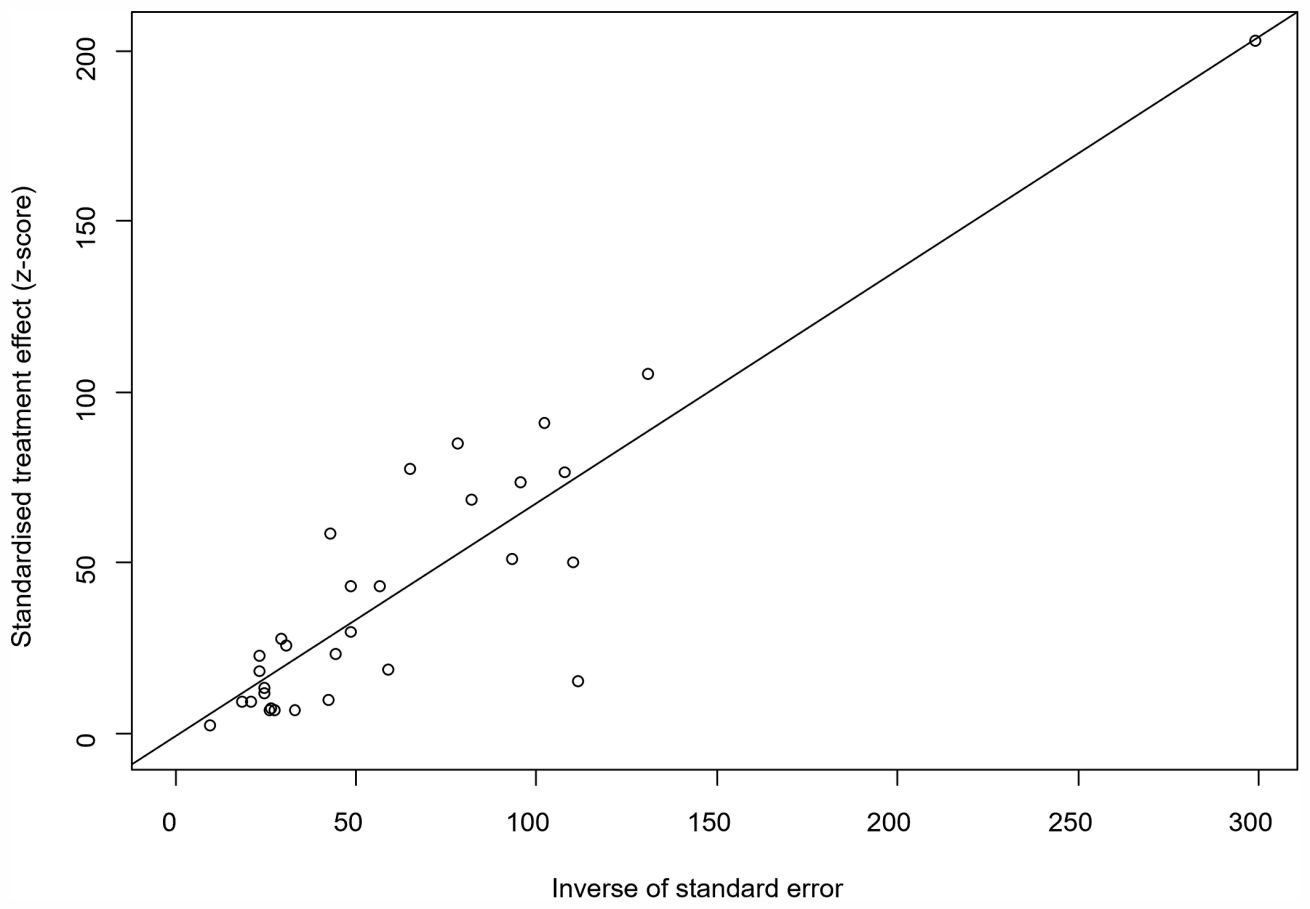
Egger’s test for publication bias.

**Figure 4:**
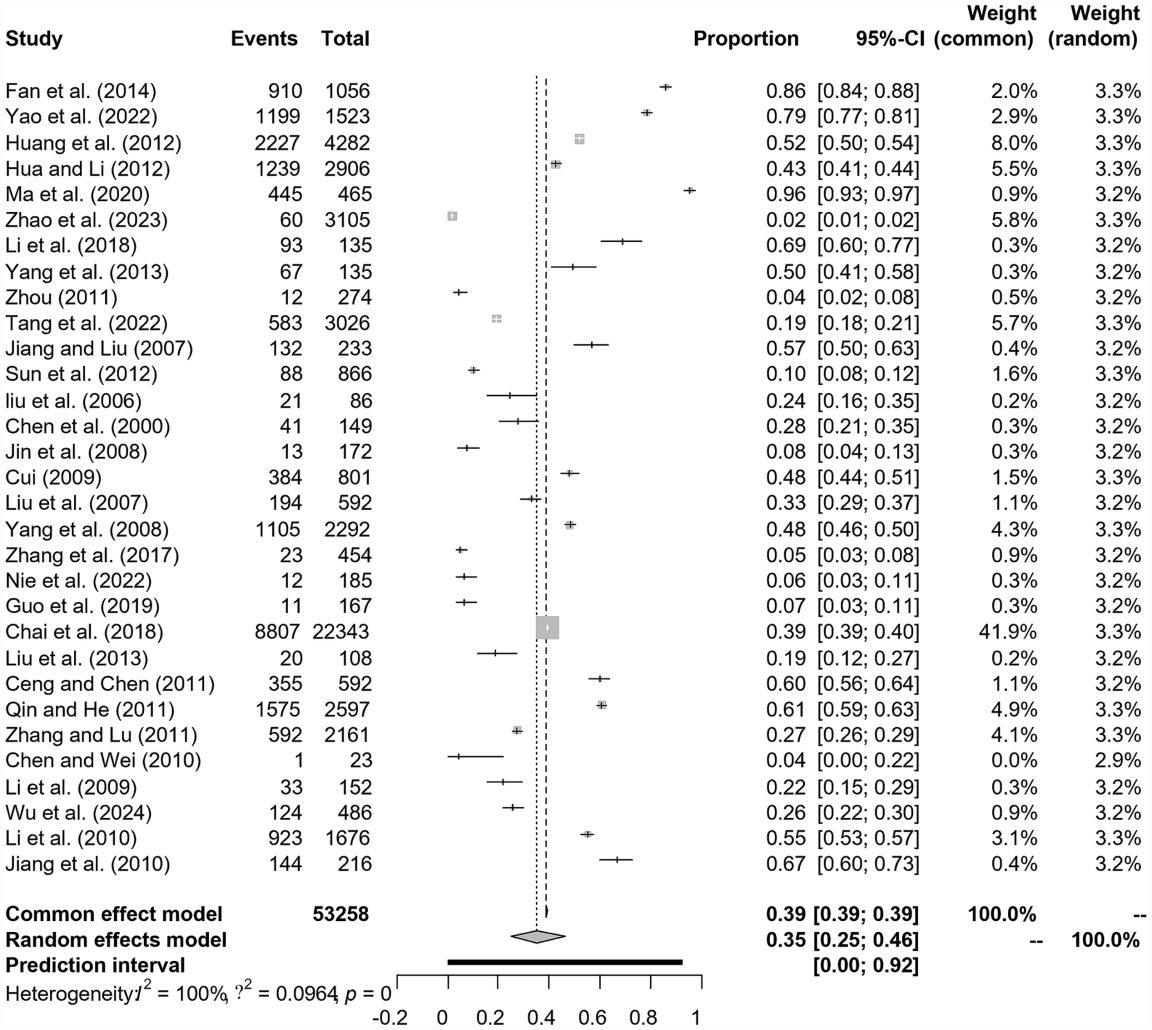
Forest plot of prevalence of epidemic encephalitis B in pig amongst studies conducted in China.

**Figure 5:**
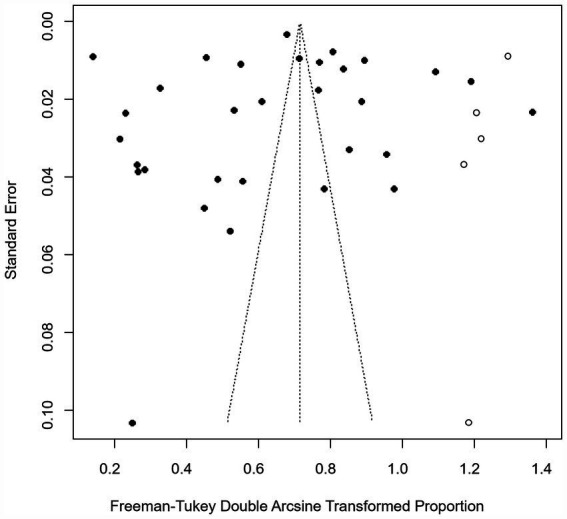
Cut-and-fill method for publication bias.

We assumed a random-effects model because there was apparent heterogeneity in the studies (*I*^2^ = 100%, *p* = 0). The extent of publication bias was assessed and illustrated by a funnel plot (Figure 2). The Egger’s test (*p* < 0.05) revealed that, there was publication bias (*p* = 0.8732, Figure 3). The heterogeneity results were shown by the forest plot (Figure 4). The result of the trim and filled analysis showed that, no trimming was performed, and no data was changed, which meant there may be no significant publication bias. Therefore, our pooled estimates were relatively robust (*p* = 0, Figure 5; Supplementary Tables S3, S4). The publication bias should be interpreted with caution because of the inconsistency in the results.

### 3.2 Sensitivity analysis results

**Figure 6:**
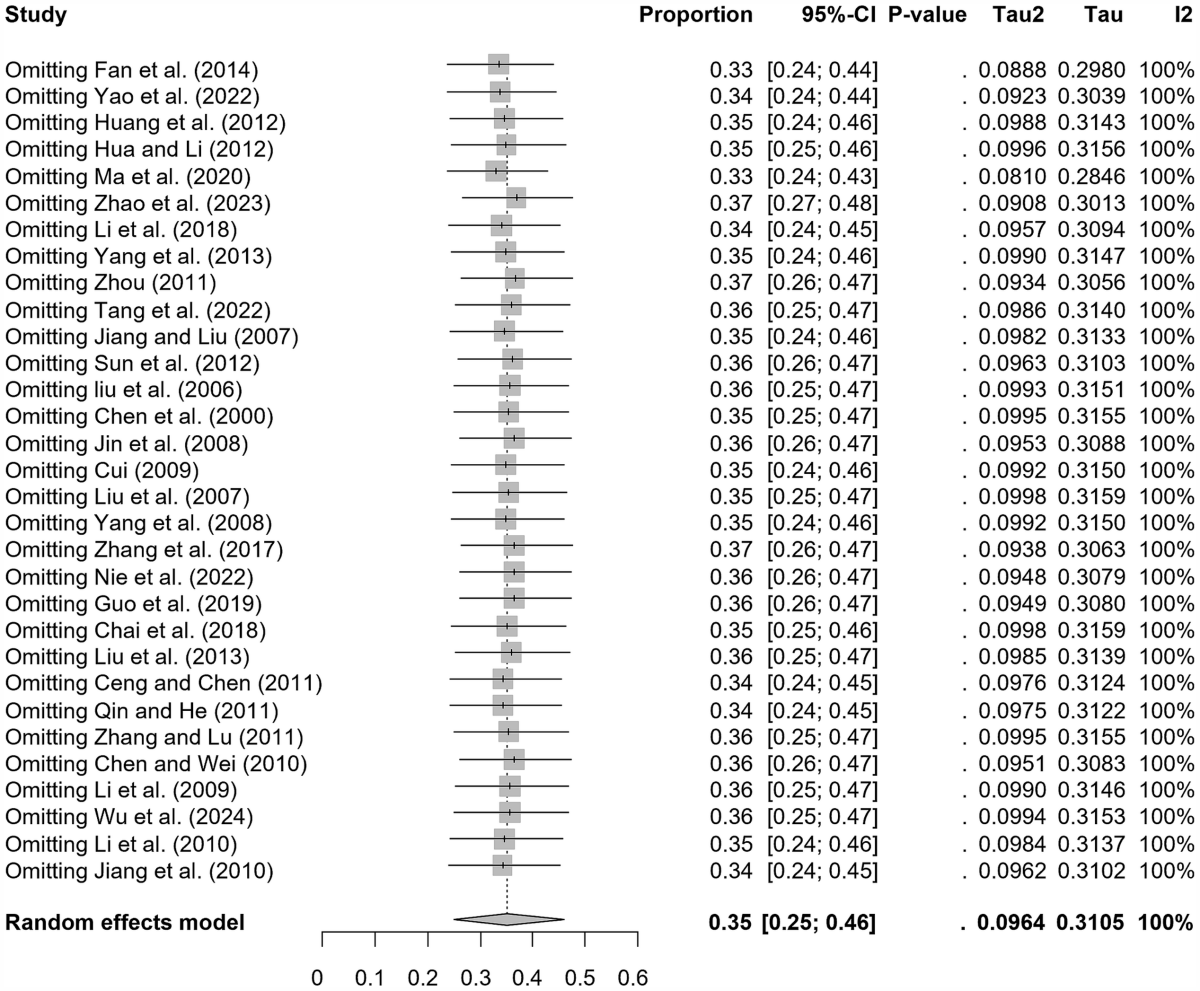
Sensitivity analysis.

Sensitivity analyses showed that, excluding any single study did not change the overall results, which remained consistent with prior analyses (Figure 6). Therefore, the findings of this review and meta-analysis were robust and reliable.

### 3.3 A meta-analysis of Japanese encephalitis in pigs in China

In China, all provinces showed a high prevalence of JE, except for Qinghai, Tibet, and Xinjiang, which were unaffected regions (45). Our meta-analysis covered seven geographic subregions: East China, South China, North China, Central China, Southwest China, Northwest China, and Northeast China. The overall prevalence of JE in the national swine population was 35.2% (95% CI: 25.1–46.1; Table 2). South China had the highest prevalence among regions at 43.8% (95% CI: 21.6–67.4; Table 2) (Supplementary Figure S1). Jiangxi Province had the highest prevalence at 86.0% (95% CI: 24.8–100.0; Table 3) (Supplementary Figure S10), followed by Chongqing Municipality at 77.4% (95% CI: 71.1–83.2; Table 3) (Supplementary Figure S10).

**Table 2:** Pooled prevalence of Japanese encephalitis of swine in Mainland China.

	No. studies	No. tested	No. positive	% (95% CI*)	Heterogeneity			Univariate meta-regression	
					*χ* ^2^	*p*-value	*I*^2^ (%)	*p*-value	Coefficient (95% CI)
Region*									
Central China	5	6,663	2,687	38.6% (16.0–64.2)	1,190.86	<0.01	99.4%		
Eastern China	4	1,683	423	31.4% (5.1–66.9)	744.48	<0.01	99.6%		
Northeastern China	4	4,149	709	7.4% (0.3–21.9)	476.92	<0.01	99.4%	0.0402	−0.3149 (−0.6156 to −0.0141)
Northern China	3	625	59	9.3% (7.1–11.8)	1.69	0.43	0.0%		
Northwestern China	4	2,896	1,404	38.2% (6.0–78.1)	2,327.27	0.00	99.9%		
Southern China	8	9,837	5,348	43.8% (21.6–67.4)	1,173.96	<0.01	99.4%		
Southwestern China	10	12,822	4,873	26.7% (17.4–37.2)	1,969.00	0.00	99.0%		
Sampling years									
2010 ago	25	10,582	4,280	36.2% (27.1–45.9)	1,740.87	0.00	98.6%		
2010–2015	8	25,567	11,236	63.4% (44.2–80.6)	2,187.10	0.00	99.7%		
2015 late	6	3,839	125	7.8% (3.4–13.5)	75.71	<0.01	93.4%	0.0003	−0.4193 (−0.6484 to −0.1902)
Sample									
Organization	1	23	1	4.4% (0.0–17.7)	0.00	–	–		
Brain tissue	2	1,120	78	11.0% (1.7–26.5)	16.61	<0.01	94.0%		
Serum	27	51,783	20,153	38.1% (27.9–48.9)	8,032.47	0.00	99.7%	0.0148	0.3637 (0.0713–0.6562)
Blood	1	167	11	6.6% (3.3–10.9)	0.00	–	–		
Detection method*									
ELISA	16	15,152	6,435	38.2% (24.5–52.9)	3,897.70	0.00	99.6%		
PCR	7	6,725	1,072	8.5% (0.6–23.2)	2,405.11	0.00	99.8%	0.0056	−0.3277 (−0.5595 to −0.0959)
RT-RAA	1	185	12	6.5% (3.3–10.5)	0.00	–	–		
LAT	9	7,403	2,909	32.4% (19.8–46.5)	805.92	<0.01	99.0%		
Breeding mode									
Farm	18	43,333	16,971	40.9% (26.4–56.3)	7,751.66	0.00	99.8%		
Free range	10	5,328	2,736	35.8% (14.9–59.7)	1,517.22	<0.01	99.4%	0.7430	−0.0451 (−0.3145 to 0.2243)
Season*									
Spring	6	1,230	258	27.5% (10.8–48.1)	180.84	<0.01	97.2%		
Winter	3	251	131	51.3% (13.6–88.2)	50.26	<0.01	96.0%		
Autumn	6	1,564	565	23.8% (5.4–49.3)	1,011.42	<0.01	99.5%	0.4227	−0.1292 (−0.4450 to 0.1866)
Summer	10	3,084	1,464	36.6% (15.3–60.9)	1,342.69	<0.01	99.3%		
Gender									
Female	9	4,680	2,753	50.0% (26.8–73.3)	1,451.80	<0.01	99.4%		
Male	8	804	303	40.6% (19.4–63.7)	365.72	<0.01	98.1%	0.5847	−0.0925 (−0.4243 to 0.2393)
Age									
Nursery pigs	9	3,219	1,078	31.2% (14.6–50.8)	1,140.24	<0.01	99.3%	0.1938	−0.1826 (−0.4581 to 0.0929)
Weaned piglets	5	1,530	360	48.4% (14.3–83.4)	361.13	<0.01	98.9%		
Fattening pigs	10	5,953	2,783	49.7% (29.8–69.7)	1,522.45	<0.01	99.4%		
Quality level									
0–2	7	27,735	11,342	29.3% (11.4–51.2)	1,652.05	0.00	99.6%		
3–4	24	25,523	9,636	35.1% (24.2–46.9)	7,212.33	0.00	99.7%	0.6245	0.0628 (−0.1887 to 0.3144)
Total	31	53,258	20,978	35.2% (25.1–46.1)	10,151.33	0.000	99.7%		

CI*: Confidence interval.

Region*: Central China: Hubei; Eastern China: Zhejiang; Northeastern China: Heilongjiang, Jilin, Liaoning; Northern China: Inner Mongolia; Northwestern China: Ningxia, Qinghai, Xinjiang.

Method*: ELISA: Enzyme linked immunosorbent assay; PCR: Polymerase Chain Reaction; RT-RAA: Reverse Transcription Recombinase Aided Amplification; LAT: Latex agglutination test.

Season*: Spring: Mar to May; Summer: Jun to Aug.; Autumn: Sep to Nov; Winter: Dec to Feb.

**Table 3:** Estimated pooled seroprevalence of Japanese encephalitis by provincial regions in China.

Province	No. Studies	Region	No. tested	No. positive	% Prevalence	% (95% CI)
Beijing	1	North China	172	13	7.5%	4.0–12.0
Fujian	2	East China	284	134	48.0%	11.5–85.8
Gansu	2	Northwest China	1,756	1,331	68.4%	45.3–87.5
Guangdong	3	Southern China	4,603	2,424	62.1%	35.6–85.2
Guangxi	4	Southern China	7,148	4,081	41.5%	12.2–74.6
Guizhou	2	Southwest China	3,498	1,594	51.2%	34.4–67.9
Hainan	2	Southern China	348	256	67.0%	0.0–100.0
Hebei	1	North China	365	35	9.6%	6.8–12.8
Henan	3	Central China	824	385	23.9%	0.0–73.3
Heilongjiang	3	Northeast China	1,286	110	7.7%	3.4–13.3
Hubei	1	Central China	30	0	0.0%	0.0–100.0
Hunan	1	Central China	3,026	583	19.3%	17.9–20.7
Jilin	3	Northeast China	2,251	487	7.0%	0.0–30.9
Jiangsu	1	East China	363	170	47.8%	41.7–52.0
Jiangxi	2	Southern China	301	213	86.0%	24.8–100.0
Liaoning	2	Northeast China	363	82	9.1%	0.0–52.0
Inner Mongolia	3	Northwest China	1,289	50	34.0%	27.4–100.0
Shandong	1	East China	832	14	1.7%	0.9–2.6
Shaanxi	1	Northwest China	188	64	34.0%	27.4–41.0
Shanghai	1	East China	152	33	21.7%	15.5–28.6
Sichuan	3	Southwest China	1,263	330	20.4%	6.7–38.9
Xizang	1	Southwest China	454	23	5.1%	3.2–7.3
Yunnan	5	Southwest China	2,895	1,225	26.9%	10.7–47.1
Chongqing	1	Southwest China	186	144	77.4%	71.1–83.2

In this study, subgroup analyses were conducted based on sampling time, region, season, testing method, age, province, sex, breeding mode, sample type, and quality score. Sampling time, region, testing method, and sample type were identified as significant risk factors for *JEV* infection in pigs (*p* < 0.05, Table 2). The prevalence of JE was 63.4% (95% CI: 44.2–80.6; Table 2) (Supplementary Figure S2) with studies conducted between 2010 and 2015 were higher than other periods. The infection rate in South China was 43.8% (95% CI: 21.6–67.4; Table 2) (Supplementary Figure S1), which was higher than the other regions. While the lowest rate in the northeast was recorded in 7.4%, (95%CI: 0.3–21.9; Table 2). In the climate subgroup, the prevalence in temperate monsoon climates was 12.7% (95% CI: 5.7–21.9; Table 2) (Supplementary Figure S19) compared to 5.1% (95% CI: 3.2–7.3; Table 2) (Supplementary Figure S19) in highland alpine regions. Within the testing method subgroup, the prevalence using ELISA was 38.2% (95% CI: 24.5–52.9; Table 2) (Supplementary Figure S4), while RT-RAA had the lowest prevalence rate in 6.5% (95%CI: 3.3–10.5; Table 2) (Supplementary Figure S4). The prevalence among samples tested as serum was 38.1% (95% CI: 27.9–48.9; Table 2) (Supplementary Figure S3). The prevalence of porcine JE in the assay method subgroups ranged from 38.2% (95% CI: 24.5–52.9; Table 2) to 6.5% (95% CI: 3.3–10.5). Among all sample types, serological testing samples had the highest prevalence of 38.1% (95% CI: 27.9–48.9; Table 2), whereas tissue samples had the lowest prevalence (4.4, 95% CI: 0.0–17.7; Table 2). In the seasonal subgroups, winter had the highest prevalence of 51.3% (95% CI: 13.6–88.2; Table 2) and autumn had the lowest prevalence of 23.8% (95% CI: 5.4–49.3; Table 2) (Supplementary Figure S6). Among the age subgroups, fattening pigs were more affected, with a prevalence of 49.7% (95% CI: 29.8–69.7; Table 2), meanwhile nursery pigs had the lowest prevalence of 31.2% (95% CI: 14.6–50.8) (Supplementary Figure S8). Among the sex subgroups, the prevalence was higher in saws (50, 95%CI: 26.8–73.3; Table 2) than in boars (40.6, 95%CI: 19.4–63.7; Table 2) (Supplementary Figure S7). Among the different farming modes, the positive detection rate was significantly higher in mass culture (41.0, 95% CI: 26.4–56.3; Table 2) than in free-range mode (35.8, 95% CI: 14.9–59.7; Table 2) (Supplementary Figure S5). In the quality score subgroup, the prevalence of score 3–4 (35.1, 95% CI: 24.2–46.9; Table 2) was higher than 0–2 (29.3, 95% CI: 11.4–51.2) (Supplementary Figure S9).

In addition, geographic factors were analyzed to further investigate the risk factors for the prevalence of porcine JE, such as latitude, longitude, rainfall, altitude, climate, and temperature variation. In the northern latitude subgroup, the highest prevalence was found at 20–30 degrees north latitude (44.8, 95% CI: 32.4–57.4; Table 4), whereas the lowest prevalence was found at 40–50 degrees north latitude (10.9, 95% CI: 10.1–11.7; Table 4). In the east longitude subgroup, the prevalence was higher in the 90–110 degree longitude range compared to the other two groups (49.5, 95% CI: 48.7–50.4; Table 4) (Supplementary Figure S12). In the altitude subgroup, the prevalence of positive detections was higher in the altitude range 0–1,000 (47.1, 95% CI: 25.8–68.9; Table 4) than in the range 4,000–15,000 (21.7, 95% CI: 6.2–43.1; Table 4) (Supplementary Figure S13). The highest positive detection rate was observed at rainfall levels of 150–200 (63.7, 95% CI: 17.1–98.1; Table 4) compared to 0–50 (24.9, 95% CI: 10.4–43.1; Table 4) (Supplementary Figure S14), and the highest prevalence rate was observed in the humidity subgroups of 75–85% at 46.6% (95% CI: 45.9–47.3; Table 4), while the lowest prevalence was observed at 40–65% (12.4, 95% CI: 4.5–23.2; Table 4) (Supplementary Figure S15). In the temperature subgroup, the highest prevalence of 53.7% (95% CI: 34.7–72.2; Table 4) was observed when the temperature reached 20–25°C, while the lowest prevalence of 14.8% (95% CI: 5.7–27.2; Table 4) (Supplementary Figures S16–118) was observed when the temperature was ranged from 0 to 10°C. The prevalence was highest when the temperature reached 20–25°C, while the lowest prevalence was observed when the temperature was 0–10°C (95% CI: 5.7–27.2; Table 4) (Supplementary Figures S16–118).

Heterogeneity across subgroups was explained by the assay method (covariate) (range 0–79.25%; R^2^-method) and geographic region (covariate) (range 60.97–97.04%; R^2^-country).

**Table 4:** Pooled prevalence of Japanese encephalitis of swine in Mainland China.

	No. studies	No. tested	No. positive	% (95% CI*)	Heterogeneity			Univariate meta-regression	
					*χ* ^2^	*p*-value	*I*^2^ (%)	*p*-value	Coefficient (95% CI)
Latitude*									
20–30	27	21,555	9,994	44.8% (32.4–57.7)	4,135.20	0.00	99.4%		
30–40	17	5,581	2,472	30.9% (17.8–45.8)	2,613.83	0.00	99.4%		
40–50	12	5,554	764	10.9% (10.1–11.7)	808.15	<0.01	98.6%	0.0001	−0.3923 (−0.5901 to −0.1945)
Longitude									
90–110	26	14,525	7,318	37.8% (27.6–48.6)	3,126.16	0.00	99.2%		
110–120	21	14,113	5,200	34.8% (19.0–52.5)	4,811.60	0.00	99.6%		
120–130	9	4,052	712	9.2% (2.9–18.3)	538.56	<0.01	98.5%	0.0044	−0.3394 (−0.5728 to −0.1061)
Altitude									
0–1,000	14	9,463	3,867	47.1% (25.8–68.9)	2,254.03	0.00	99.4%		
1,000–4,000	22	14,504	5,639	22.8% (12.7–34.7)	4,314.25	0.00	99.5%	0.0915	−0.1580 (−0.3416 to 0.0255)
4,000–15,000	7	2,441	607	21.7% (6.2–43.1)	1,122.04	<0.01	99.5%		
15,000–20,000	10	5,156	2,826	36.6% (21.5–53.1)	1,192.30	<0.01	99.2%		
20,000–40,000	3	1,126	291	32.8% (1.4–79.1)	380.70	<0.01	99.5%		
Rainfall									
0–50	9	3,139	1,519	24.9% (10.4–43.1)	1,680.95	0.00	99.5%		
50–100	32	15,097	4,825	28.9% (18.9–40.1)	4,944.07	0.00	99.4%	0.5177	−0.0611 (−0.2462 to 0.1240)
100–150	7	7,757	3,306	28.5% (11.9–48.9)	1,633.49	0.00	99.6%		
150–200	5	4,762	2,596	63.7% (17.1–98.1)	657.45	<0.01	99.4%		
200–350	3	1,935	984	33.2% (13.8–56.1)	100.46	<0.01	98.0%		
Humidity									
40–65	17	7,861	2,025	12.4% (4.5–23.2)	4,376.41	0.00	99.6%		
65–70	7	25,885	760	23.6% (12.3–37.2)	242.76	<0.01	97.5%		
70–85	32	22,244	10,445	45.1% (33.9–56.6)	3,785.25	0.00	99.2%	<0.0001	0.3318 (0.1685–0.4952)
Minimum annual temperature									
−10 to 0	2	958	98	10.1% (8.2–12.1)	0.1	0.75	0.0%		
0–10	15	7,166	2,097	14.4% (5.4–26.6)	3,680.14	0.00	99.6%	0.0047	−0.2791 (−0.4727 to −0.0855)
10–20	31	17,406	7,328	39.3% (28.6–50.5)	4,278.96	0.00	99.3%		
20–30	8	7,160	2,876	43.5% (16.9–72.2)	809.32	<0.01	99.1%		
Maximum annual temperature									
0–10	2	1,622	480	20.4% (5.0–42.5)	20.94	<0.01	95.2%		
10–20	22	12,530	4,533	20.0% (10.2–32.0)	5,056.62	0.00	99.6%		
20–30	32	18,538	8,217	40.7% (29.2–52.6)	4,967.92	0.00	99.4%	0.0116	0.2264 (0.0506–0.4022)
Average annual temperature									
0–10	14	7,691	2,118	14.8% (5.7–27.2)	3,701.81	0.00	99.6%		
10–15	5	3,931	1,671	24.1% (4.6–52.3)	328.44	<0.01	98.8%		
15–20	25	10,361	3,469	32.9% (21.3–45.7)	2,721.66	0.00	99.1%		
20–25	12	10,707	5,972	53.7% (34.7–72.2)	1,261.55	<0.01	99.1%	0.0068	0.2897 (0.0797–0.4998)
Climate									
Oceanic subtropical monsoon climate	8	7,133	3,648	43.7% (21.7–67.0)	404.63	<0.01	98.3%		
Plateau alpine climate	1	454	23	5.1% (3.2–7.3)	0.00	–	–		
Subtropical mild monsoon climate	3	3,207	1,452	73.2% (25.9–99.9)	263.54	<0.01	99.2%		
Subtropical monsoon climate	23	12,222	5,280	36.2% (26.1–46.9)	2,770.30	0.00	99.2%		
Temperate continental climate	3	1,944	1,395	57.2% (31.1–81.3)	175.54	<0.01	98.9%		
Temperate continental monsoon climate	5	2,273	46	2.5% (0.2–6.6)	30.50	<0.01	86.9%		
Temperate monsoon climate	11	5,109	1,130	12.7% (5.7–21.9)	818.47	<0.01	98.8%	0.0110	−0.2843 (−0.5035 to −0.0652)
Tropical monsoon Marine climate	2	348	256	66.9% (0.0–100.0)	317.14	<0.01	99.7%		

CI*: Confidence interval.

## 4 Discussion

Porcine JE is a zoonotic infectious disease, that affects both humans and animals. Geographically, it is endemic in regions in the Far East, South, and Southeast Asian countries (46, 47) including South Korea, Thailand, Java (Indonesia), and the Primrosy region of Siberia (Russia), and in Kerala, and Haryana, India (48, 49). Recently, cases of JE have also been reported in mainland Australia, Guam, and USA (50). Surprisingly, the morbidity and mortality rates due to infection with JE have increased in China, except in Northern, Northeast China, Qinghai, Xinjiang, and Tibet. Meanwhile, the prevalence of *JEV* is rising globally in endemic areas, posing a serious threat to public health and the livestock industry (51). Pigs are intermediate hosts for the *JEV*, whereas humans are the final hosts, and the infected carrier pigs are the primary source of transmission. Clinically, the disease leads to abortion, stillbirth, mummified fetuses in sows, and testicular inflammation in boars (25). This is clearly reflecting the expanding range of the disease’s endemicity, and posing a growing public health concern (32).

To our knowledge, this is the first meta-analysis on the prevalence of porcine JE in China. The findings of this study could inform actionable control measures to improve animal husbandry practices. The analysis of the obtained results revealed, significant variations in the prevalence of JE in pigs across regions, sampling periods, and breeding practices (*p* < 0.05). The national swine population showed an overall prevalence rate of 35.2% for JE (Table 2). At the regional level, the South China showed a high significant (*p* < 0.05) prevalence (43.8, 95% CI: 21.6–67.4; Table 2) compared to the other regions (Table 2). Also, Jiangxi Province had the highest prevalence, followed by Chongqing Municipality (Table 2). Both provinces are located within the subtropical monsoon climate zone, and characterized by hot summers, mild winters, four distinct seasons, and a well-developed monsoon pattern, all of which are likely to influence the spreading of the disease. Numerous analyses have indicated that the incidence of *JEV* infection has a seasonal pattern and closely related to geographical distribution, and climate (19). Study in southwest China found significant associations between JE incidence and agricultural and climatic variables, including monthly precipitation and monthly mean minimum and maximum temperatures (52). This climate provides favorable conditions for its spread. The region’s average annual temperature ranges from 20°C to 25°C. This warm climate promotes the reproduction and transmission of vector organisms, such as mosquitoes. Consequently, swine populations in the subtropical region face a higher risk of infection, leading to elevated prevalence rates. Furthermore, the higher elevations, cold and arid climate, and low annual precipitation in the western region are unfavorable conditions for mosquito survival and reproduction, leading to weaker transmission of JE. Meanwhile, the low elevation, abundant plains, high precipitation, and vegetation of South China create optimal conditions for mosquito proliferation, thereby facilitating the local spreading of *JEV* (53–55).

The prevalence of JE between 2010 and 2015 was 63.4%, that was higher than in other periods. A total of 858 pig serum samples from both large-scale and rural free-range farms in Longyan City, Fujian Province, were tested for *JEV* antibody levels between 2011 and 2014. The elevated JE prevalence from 2010 to 2015 was influenced by several factors. A substantial research has consistently demonstrated a significant positive correlation between increasing temperatures and both the proliferation of mosquito populations and elevated incidence of mosquito-borne diseases (56). Average annual precipitation of 100–150 millimeters and temperatures between 15 and 20°C fostered mosquito proliferation, correlated positively with JE incidence and leading to a rise in in infected cases. Distinct climatic subtypes within temperate regions showed varying JE prevalence patterns. In Gansu Province, China, the cases appeared in a temperate arid climate, indicating a possible spread to new areas (57). In temperate zones, the disease transmission is typically epidemic and seasonal, with most cases occurring during summer months (58). This contrasts with subtropical and tropical regions where transmission can occur year-round, peaking during the rainy season (58). The seasonal nature of JE in temperate areas limits the overall prevalence compared to regions with continuous transmission (59). Serological testing revealed that, the prevalence of *JEV* in immunized pigs from large-scale and free-range farms were 72.17 and 57.72%, respectively. In comparison, the seropositivity rate in immunized pigs was 69.71%, slightly higher than the 68.89% in unimmunized pigs (60). Significant differences were observed between the two cases, and due to the divergent objectives of the studies, investigations involving immunized pigs were excluded from our analysis, while only studies utilizing non-immunized pigs were included. The JE remains a serious concern in Fujian Province and requires continued attention. One of the included articles showed that, 78 porcine *JEV* nucleic acids were detected in 263 samples collected from 14 different swine farms in the south from 2011 to 2018, with a positivity rate of 29.7% (61). The emergence of this cause may be due to the location in the tropics and subtropics, where the warm and humid climate, the high density of mosquitoes, and the large number of domestic pigs provide the natural conditions for the spread and reproduction of *JEV* (22).

Various methods have been used in epidemiological studies of *JEV*, including virus isolation, RT-PCR, RT-qPCR, and microdroplet digital PCR (ddPCR) (13). Virus isolation is a time-consuming, labor-intensive process that often taking over a week to complete, this limits its use in large-scale epidemiologic investigations. The serum neutralization test (SNT) is the standard method for serological detection of *JEV*, but cross-reactivity between the different *flaviviruses* within the same genus was recorded using this tool which reflects the inaccurate results (62). On the other side, the previously mentioned molecular techniques usually take 2–3 h for completion (63–65). False positivity varies depending on the used tools and could affect the accurate estimation of the disease prevalence. Accordingly, four major detection methods for JE were usually applied including; ELISA, PCR, RT-RAA, and LAT. ELISA is a fundamental technique in immunology and molecular biology, utilizing antigen–antibody binding with enzymatic and colorimetric assays for quantitative analysis of target molecules. It detects and quantifies specific proteins, peptides, antibodies, or antigens in biological samples, making it essential in research and diagnostics (66). This technique is extensively used to detect antibodies and antigens for diagnosing and *JEV* monitoring but is prone to cross-reactivity with other flaviviruses like *yellow fever virus*, which can lead to false results and affect prevalence estimates. To address this issue more effectively, it is suggested to develop more specific detection methods for antigen, including secondary screening alongside PCR assays or alternative immunological detection techniques in future studies to mitigate the impact of cross-reactivity. PCR utilizes the semi-conservative replication of DNA for *in vitro* enzymatic synthesis and amplification of specific nucleic acid sequences. The specificity of this technique is achieved through the utilization of oligonucleotide primers complementary to the flanking regions of the target sequence (67). RT-PCR involves the conversion of mRNA into cDNA utilizing reverse transcriptase, which subsequently serves as the template for amplifying the target fragment. The RNA template employed in this procedure may comprise total RNA, mRNA, or in vitro transcribed RNA (68). LAT is an indirect agglutination assay using latex particles as carriers. Soluble antigens are adsorbed on these particles, allowing specific antibodies to bind and promote agglutination (69). It was found that, ELISA was significantly (*p* = 0.0056, Table 5) the commonly used tool. It offers several advantages, including rapidity, high efficiency, low cost, specificity, high sensitivity, simplicity, and no need for high aseptic procedures. Also, it enables the simultaneous testing of multiple serum samples (70). Given the large pig population, rapid turnover, and high infection rates of *JEV* in the country (71), the specificity, reproducibility, and operational simplicity of ELISA render it an optimal method for the detection of porcine JE antibodies due to infection adding to the evaluation of antibody titers following immunization (70). It is noteworthy that, some studies did not explain whether the pigs had been immunized with swine JE vaccine or not. So, false-positive results contribute to heterogeneity in the results (72).

**Table 5:** Included studies of Japanese encephalitis of swine in Mainland China.

Reference ID	Sampling time	Detection method	No. tested	No. positive	Prevalence	Study design	Score
Central China							
Tang et al. (2022)	2019–2021	ELISA	3,026	583	0.192664	Cross sectional	3
Cui (2009)	2008–2009	ELISA	801	384	0.4794	Cross sectional	3
Chai et al. (2018)	2006–2012	UN	2,597	1,575	0.606469	Cross sectional	2
Chen and Wei (2010)	2007.6–2008.9	RT-PCR	23	1	0.043478261	Cross sectional	4
Jiang et al. (2010)	2008–2009	ELISA	216	144	0.6666667	Cross sectional	3
East China							
Fan et al. (2014)	2014	ELISA	564	283	0.501773	Cross sectional	3
Zhao et al. (2023)	2016–2020	PCR	832	14	0.016827	Cross sectional	3
Li et al. (2018)	2011–2014	ELISA	135	93	0.6888889	Cross sectional	3
Li et al. (2009)	2006–2007	Other	152	33	0.217105	Cross sectional	3
North China							
Jin et al. (2008)	2006.7	ELISA	172	13	0.075581395	Cross sectional	3
Guo et al. (2019)	2015–2016	ELISA	88	11	0.065868263	Cross sectional	3
Chai et al. (2018)	2006–2012	UN	365	35	0.09589	Cross sectional	2
Northeast China							
Zhao et al. (2023)	2016–2020	PCR	1,043	29	0.027804	Cross sectional	3
Sun et al. (2012)	1997–2000	LAT	866	88	0.101616628	Cross sectional	4
Guo et al. (2019)	2015–2016	ELISA	79	0	0	Cross sectional	3
Zhang and Lu (2011)	2006–2009	ELISA	2,161	592	0.273947247	Cross sectional	3
Northwest China							
Fan et al. (2014)	2014	ELISA	188	64	0.340425532	Cross sectional	3
Yao et al. (2022)	UN	ELISA	1,523	1,199	0.787261983	Cross sectional	2
Zhao et al. (2023)	2016–2020	PCR	952	9	0.009453782	Cross sectional	3
Jiang and Liu (2007)	2006	LAT	233	132	0.566523605	Cross sectional	4
Southern China							
Fan et al. (2014)	2014	ELISA	304	108	0.355263158	Cross sectional	3
Huang et al. (2012)	2009–2011	ELISA	4,282	2,227	0.520084073	Cross sectional	3
Ma et al. (2020)	2013	ELISA	465	445	0.956989247	Cross sectional	3
Zhao et al. (2023)	2016–2020	PCR	278	8	0.028776978	Cross sectional	3
Liu et al. (2006)	2002–2003	LAT	86	21	0.244186047	Cross sectional	2
Chen et al. (2000)	2000	LAT	149	41	0.275167785	Cross sectional	2
Qin and He (2011)	2008–2010	LAT	2,597	1,575	0.606469003	Cross sectional	3
Li et al. (2010)	2008–2009	RT-PCR	1,676	923	0.55071599	Cross sectional	4
Southwest China							
Hua and Li (2012)	UN	LAT	2,906	1,239	0.426359257	Cross sectional	2
Yang et al. (2013)	2010–2012	LAT	135	67	0.496296296	Cross sectional	3
Zhou (2011)	UN	LAT	274	12	0.04379562	Cross sectional	2
Liu et al. (2007)	2002–2006	ELISA	592	194	0.327702703	Cross sectional	3
Yang et al. (2008)	2005–2007	LAT	2,292	1,105	0.482111693	Cross sectional	3
Zhang et al. (2017)	UN	ELISA	454	23	0.050660793	Cross sectional	2
Nie et al. (2022)	2020–2021	RT-RAA	185	12	0.064864865	Cross sectional	3
Liu et al. (2013)	2009–2010	RT-PCR	108	20	0.185185185	Cross sectional	3
Ceng and Chen (2011)	2010	ELISA	592	355	0.599662162	Cross sectional	3
Wu et al. (2024)	2007–2008	ELISA	486	124	0.255144033	Cross sectional	4

UN*: unclear.

LAT*: Latex agglutination test.

RT-PCR*: Reverse Transcription-Polymerase Chain Reaction.

RT-RAA*: Reverse Transcription Recombinase Aided Amplification.

PCR*: Polymerase Chain Reaction.

ELISA*: Enzyme linked immunosorbent assay.

It was recorded that; Pigs are one of the main hosts of *JEV* (73, 74). The prolonged viremia in the blood of pigs infected with the *JEV*, characterized by high viral loads and infectiousness, which could be the main source of human infection (35). Once the virus enters the host, it rapidly invades the bloodstream and replicates in internal organs such as (heart, liver, spleen, kidneys), causing brief viremia that lasts 3–7 days. The virus can cross the blood–brain barrier, invade the central nervous system, and replicate in brain tissue, causing lesions and neurological symptoms (75, 76). In the present study, our analysis of various sample types showed that serum had a higher detected prevalence compared to other tissues. Analysis of *JEV* serum data from Chinese swine herds showed that the prevalence and distribution of *JEV* in pigs also exhibited seasonal and geographic variation; *JEV* infections appeared 1–2 months earlier in southern China than in northern parts (26). These characteristics not only allow pigs to play an important role in the *JEV* transmission chain, but also provide a warning to the public health community that pigs are potential reservoirs of viruses that may directly or indirectly infect humans, especially if they have high viral loads in their blood with the ability to cross the blood–brain barrier, enter the central nervous system and replicate in brain tissue (77), causing neurological lesions that lead to clinical manifestations such as neurological symptoms, meningitis, encephalitis, and other serious diseases (11, 78).

Immunization greatly affects disease incidence in pig populations. Significant emphasis was placed on rigorous screening of unvaccinated pig herds, excluding articles that did not specify immunized populations and antibody protection rates. All included studies came from large-scale farms and free-range herds with unvaccinated pigs. According to the World Health Organization, the vaccine currently used for JE is the SA14-14-2 strain (79), and studies have shown vaccine efficacy to be between 80 and 99% after a single dose and 98% or higher after two doses (80). Therefore, for studies that did not explicitly state whether the subjects had been vaccinated, when the seropositive rate of pigs exceeded 90%, we considered the herd to be immune. For studies that did not explicitly state whether subjects had been immunized, we assumed that the seropositivity rate among pigs exceeded 90%, as vaccinated pigs generate antibodies, resulting in a higher antibody positivity rate. Through rigorous screening, we minimized immune factor confounding to accurately analyze the JE prevalence.

Surprisingly, the infection rate was higher in winter than in other seasons, though the difference was not statistically significant (Table 2). The incidence and prevalence of the disease show clear seasonality, typically peaking from July to September, then sharply declining after October. The disease is usually sporadic but can also become endemic (14). In our study, the phenomenon of higher prevalence in winter may be related to the regions included in the study. Especially in Hainan, Guangdong, and Yunnan provinces, which have warmer climates with insignificant seasonal variations, mosquitoes are active throughout the year. Therefore, even in winter, the mosquito population remains high, leading to higher infection rates in that season, which in turn may have influenced the bias of the study results. This disease peaks in prevalence during China’s rainy summer and autumn. Epidemic peaks occur from June to July in southern regions, from July to August in northern regions, and from August to September in northeastern regions. For instance, irrigated rice fields provide ideal breeding grounds for Culex tritaeniorhynchus, the primary vector for *JEV* transmission (81). Variations in environmental conditions and temperatures affect mosquito activity, leading to distinct disease transmission patterns across different areas (82). The increased precipitation during the summer and fall seasons creates more favorable breeding conditions for mosquitoes, resulting in a substantial increase in both of their population density and activity levels (57). As a consequence, this exacerbates the transmission of *JEV*. In areas with intensive rice farming and pig production, JE transmission is likely to increase due to the creation of suitable environments for vector mosquitoes and amplifying hosts (19). Studies indicate that tropical regions lack seasonality, allowing the disease to occur year-round (83). Interestingly, the same observation of high incidence rate was recorded in winter compared to the other seasons but with a different insect-born pathogen (2, 11).

The epidemiology of porcine JE is mainly driven by mosquito as the primary virus vector (84). It has a well-defined transmission route, mainly through mosquito bites, so mosquito control is a key measure to prevent disease transmission. In areas where the climate is more stable and mosquitoes are active throughout the year, especially in tropical and subtropical areas, prevention and control strategies for epidemics should focus on strengthening herd management and immunization (15). However, swine JE lacks specific antiviral treatments, so management relies on supportive care and immune enhancement. Prevention involves immunization, vector control, and managing pig populations (85). Live *JEV* vaccines are recommended in endemic or high-risk regions. Since JE transmission is linked to blood-feeding arthropods like mosquitoes, controlling these vectors by the different tools is crucial for prevention (86).

Our meta-analysis included five studies with quality scores of 4 or 5, 26 studies with scores of 2 or 3, and none with scores of 0 or 1. Our review for the moderate-quality studies revealed that several detailed descriptions of seasons, random sampling methods, and sampling procedures were lacked. Neglecting of seasonal factors may lead to seasonal bias in epidemiologic results, especially for those diseases that are strongly influenced by climatic and environmental changes, and the lack of seasonal descriptions will limit the accuracy and extrapolation of results. Lack of random sampling or poor description may then lead to sample selection bias, making the results of the study unable to truly reflect the characteristics of the target group, thus affecting the reliability and scientific value of the results. In addition, unclear details of the sampling method may lead to reduced comparability across studies, thus affecting the accuracy of meta-analyses. Therefore, it is recommended that, future researchers in the future should cover these shortages to improve the reliability of their findings. This study used regression analysis to investigate factors affecting JE spreading, identifying a significant correlation between sample size and JE prevalence. However, the analyses did not account for all potential confounding variables. Future research should include more covariates to improve generalizability and establish stronger causal relationships.

This meta-analysis has several strengths, including a broad temporal range, extensive geographic coverage, and well-defined analytical methods, but also some limitations were present. Firstly, the selected articles were limited to Chinese or English, potentially excluding relevant studies in other languages. Secondly, the articles were sourced from six databases only, which may have excluded relevant studies from other sources. Lastly, the study concentrates on specific Chinese provinces, underrepresenting regions like Qinghai, Tibet, and Xinjiang. This limited representation may impact findings and compromise external validity and robustness. Future studies should adopt a more comprehensive sampling approach, especially in underrepresented western provinces, to better assess national prevalence.

## 5 Conclusion

The current meta-analysis showed that the prevalence of JE infection in swine is widely distributed across China. Additionally, the disease is more prevalent in regions with consistently hot and humid climates. Thus, we recommend continuous surveillance of swine populations and implementing isolation measures to reduce mosquito contact with herds. Furthermore, awareness of JE should be raised in regions where the disease receives less attention, and epidemiological investigations should be promptly conducted to ensure timely control of its spread. The high prevalence of this disease swine can cause significant economic losses for farmers and herdsmen adding to increasing the risk of infection. Therefore, attention to animal welfare and application of all precaution measures to limit the spread of JE is crucial for intensive pig farming. This study lays a foundation for future research on strategies to control JE.

## Data Availability

The raw data supporting the conclusions of this article will be made available by the authors without undue reservation.

## References

[1] Niu TM Yu LJ Zhao JH Zhang RR Ata EB Wang N . Characterization and pathogenicity of the porcine epidemic diarrhea virus isolated in China. Microb Pathog. (2023) 174:105924. doi: 10.1016/j.micpath.2022.105924, PMID: 36473667

[2] Ata EB Abdel-Aziz TH Abdel-Ghany HSM Elsawy BSM Abdullah H Abouelsoued D . Molecular and serological diagnosis of the circulating Trypanosoma evansi in Egyptian livestock with risk factors assessment. Microb Pathog. (2024) 197:107073. doi: 10.1016/j.micpath.2024.107073 39454805

[3] Kasem S Yu MHH Alkhalefa N Ata EB Nayel M Abdo W . Impact of equine herpesvirus-1 ORF15 (Eul45) on viral replication and neurovirulence. Vet Microbiol. (2024) 298:110234. doi: 10.1016/j.vetmic.2024.110234, PMID: 39180797

[4] Ibrahim HS Alsenosy AA El-Ktany EM Ata EB Abas OM. Anthelmintic efficacy and pharmacodynamic effects of levamisole-oxyclozanide combination as (Levanide®) in fattening calves. Egypt J Vet Sci. (2023) 54:1245–54. doi: 10.21608/ejvs.2023.219811.1532

[5] Shalaby H Kandil O Hendawy S Elsawy BS Ashry HM El-Namaky A . Dynamics of Haemonchus contortus coproantigen appearance in feces of experimentally infected sheep. Egypt J Vet Sci. (2024) 55:1307–14. doi: 10.21608/ejvs.2024.251684.1693

[6] Ata EB Li ZJ Shi CW Yang GL Yang WT Wang CF. African swine fever virus: a raised global upsurge and a continuous threaten to pig husbandry. Microbe Pathog. (2022) 167:105561. doi: 10.1016/j.micpath.2022.105561, PMID: 35526679

[7] Hu TY Lian YB Qian JH Yang YL Ata EB Zhang RR . Immunogenicity of engineered probiotics expressing conserved antigens of influenza virus and FLIC flagellin against H9N2 AI infection in mice. Res Vet Sci. (2022) 153:115–26. doi: 10.1016/j.rvsc.2022.10.024, PMID: 36351352

[8] Sha W Beshir Ata E Yan M Zhang Z Fan H. Swine colibacillosis: analysis of the gut bacterial microbiome. Microorganisms. (2024) 12:1233. doi: 10.3390/microorganisms12061233, PMID: 38930615 PMC11205844

[9] Yang W-T Yang W Jin Y-B Ata EB Zhang R-R Huang HB . Synthesized swine influenza NS1 antigen provides a protective immunity in a mice model. J Vet Sci. (2020) 21:e66. doi: 10.4142/jvs.2020.21.e66

[10] Hao Y Sheng K Ruan WK. Expression of non-structural proteins in Japanese encephalitis virus and their interaction with host hnRNP K in Chinese. College of Animal Science and Technology, Beijing University of Agriculture (2024), 39, 37–42. doi: 10.13473/j.cnki.issn.1002-3186.2024.0108

[11] Ashraf U Ding Z Deng S Ye J Cao S Chen Z. Pathogenicity and virulence of Japanese encephalitis virus: neuroinflammation and neuronal cell damage. Virulence. (2021) 12:968–80. doi: 10.1080/21505594.2021.1899674, PMID: 33724154 PMC7971234

[12] Wang LP Yuan Y Liu YL Lu QB Shi LS Ren X . Etiological and epidemiological features of acute meningitis or encephalitis in China: a nationwide active surveillance study. Lancet Reg Health West Pac. (2022) 20:100361. doi: 10.1016/j.lanwpc.2021.100361, PMID: 35036977 PMC8743210

[13] Nie M Zhou Y Li F Deng H Zhao M Huang Y . Epidemiological investigation of swine Japanese encephalitis virus based on RT-RAA detection method. Sci Rep. (2022) 12:9392. doi: 10.1038/s41598-022-13604-4, PMID: 35672440 PMC9172605

[14] Li F Li H Yang L Wang L Gu L Zhong G . The spatial-temporal pattern of Japanese encephalitis and its influencing factors in Guangxi, China. Infect Genet Evol. (2023) 111:105433. doi: 10.1016/j.meegid.2023.105433, PMID: 37037290

[15] Wang Q Yang S Yang K Li X Dai Y Zheng Y . CD4 is an important host factor for Japanese encephalitis virus entry and replication in PK-15 cells. Vet Microbiol. (2023) 287:109913. doi: 10.1016/j.vetmic.2023.109913, PMID: 38006719

[16] Paul KK Sazzad HMS Rahman M Sultana S Hossain MJ Ledermann JP . Hospital-based surveillance for Japanese encephalitis in Bangladesh, 2007–2016: implications for introduction of immunization. Int J Infect Dis. (2020) 99:69–74. doi: 10.1016/j.ijid.2020.07.026, PMID: 32721530 PMC7566160

[17] Impoinvil DE Baylis M Solomon T. Japanese encephalitis: on the one health agenda. Curr Top Microbiol Immunol. (2013) 365:205–47. doi: 10.1007/82_2012_243, PMID: 22886540

[18] De Y Zou WZ Liu H. Overview of porcine epidemic encephalitis B and its prevention and treatment in pigs. Chinese Livestock Poultry Breed Chinese. (2022) 18:138–40.

[19] Erlanger TE Weiss S Keiser J Utzinger J Wiedenmayer K. Past, present, and future of Japanese encephalitis. Emerg Infect Dis. (2009) 15:1–7. doi: 10.3201/eid1501.080311, PMID: 19116041 PMC2660690

[20] Ricklin ME García-Nicolás O Brechbühl D Python S Zumkehr B Nougairede A . Vector-free transmission and persistence of Japanese encephalitis virus in pigs. Nat Commun. (2016) 7:10832. doi: 10.1038/ncomms10832, PMID: 26902924 PMC4766424

[21] Liu QL Jian WX Shi CQ Xia ZH Yang GY Hong J . Monitoring immune antibodies against Japanese encephalitis in pigs from a large-scale farm in Yuping County, Guizhou Province from 2020 to 2022 (in Chinese). Animals Breed Feed. (2024) 23:75–8. doi: 10.13300/j.cnki.cn42-1648/s.2024.11.016

[22] Van den Hurk AF Ritchie SA Mackenzie JS. Ecology and geographical expansion of Japanese encephalitis virus. Annu Rev Entomol. (2009) 54:17–35. doi: 10.1146/annurev.ento.54.110807.090510, PMID: 19067628

[23] Simpson DI Smith CE Marshall TF Platt GS Way HJ Bowen ETW . Arbovirus infections in Sarawak: the role of the domestic pig. Trans R Soc Trop Med Hyg. (1976) 70:66–72. doi: 10.1016/0035-9203(76)90010-9, PMID: 1265821

[24] Wang H Li Y Liang X Liang G. Japanese encephalitis in Mainland China. Jpn J Infect Dis. (2009) 62:331–6. PMID: 19762980

[25] Mansfield KL Hernández-Triana LM Banyard AC Fooks AR Johnson N. Japanese encephalitis virus infection, diagnosis and control in domestic animals. Vet Microbiol. (2017) 201:85–92. doi: 10.1016/j.vetmic.2017.01.014, PMID: 28284628

[26] Chai C Wang Q Cao S Zhao Q Wen Y Huang X . Serological and molecular epidemiology of Japanese encephalitis virus infections in swine herds in China, 2006–2012. J Vet Sci. (2018) 19:151–5. doi: 10.4142/jvs.2018.19.1.151, PMID: 28693301 PMC5799393

[27] Park SL Huang YS Vanlandingham DL. Re-examining the importance of pigs in the transmission of Japanese encephalitis virus. Pathogens. (2022) 11:575. doi: 10.3390/pathogens11050575, PMID: 35631096 PMC9146973

[28] Lyons AC Huang YS Park SL Ayers VB Hettenbach SM Higgs S . Shedding of Japanese encephalitis virus in oral fluid of infected swine. Vector Borne Zoonotic Dis. (2018) 18:469–74. doi: 10.1089/vbz.2018.2283, PMID: 29742002

[29] Zhang XX Ren WX Tan QD Hou GY Fei YC Zhao LJ . Meta-analysis of toxoplasma gondii in pigs intended for human consumption in Mainland China. Acta Trop. (2019) 198:105081. doi: 10.1016/j.actatropica.2019.105081, PMID: 31299285

[30] Banerjee S Sen Gupta PS Bandyopadhyay AK. Insight into SNPs and epitopes of E protein of newly emerged genotype-I isolates of JEV from Midnapur, West Bengal, India. BMC Immunol. (2017) 18:13. doi: 10.1186/s12865-017-0197-9, PMID: 28264652 PMC5339996

[31] Ning-Qing C. Control of arboviral encephalitis in China (Author's Transl). Med Trop (Mars). (1980) 40:555–9. 6255282

[32] Yuan L Wu R Liu H Wen X Huang X Wen Y . Tissue tropism and molecular characterization of a Japanese encephalitis virus strain isolated from pigs in Southwest China. Virus Res. (2016) 215:55–64. doi: 10.1016/j.virusres.2016.02.001, PMID: 26851509

[33] WHO. Japanese encephalitis vaccines: who position paper, February 2015–recommendations. Vaccine. (2016) 34:302–3. doi: 10.1016/j.vaccine.2015.07.057, PMID: 26232543

[34] Li YX Li MH Fu SH Chen WX Liu QY Zhang HL . Japanese encephalitis, Tibet, China. Emerg Infect Dis. (2011) 17:934–6. doi: 10.3201/eid1705.101417, PMID: 21529419 PMC3321773

[35] Weaver SC Barrett AD. Transmission cycles, host range, evolution and emergence of arboviral disease. Nat Rev Microbiol. (2004) 2:789–801. doi: 10.1038/nrmicro1006, PMID: 15378043 PMC7097645

[36] Ni HB Gong QL Zhao Q Li XY Zhang XX. Prevalence of Haemophiles parasuis "Glaesserella Parasuis" in pigs in China: a systematic review and meta-analysis. Prev Vet Med. (2020) 182:105083. doi: 10.1016/j.prevetmed.2020.105083, PMID: 32652336

[37] Moher D Liberati A Tetzlaff J Altman DG. Preferred reporting items for systematic reviews and meta-analyses: the PRISMA statement. Ann Intern Med. (2009) 6:e1000097. doi: 10.1371/journal.pmed.1000097, PMID: 19622511

[38] Moher D Shamseer L Clarke M Ghersi D Liberati A Petticrew M . Preferred reporting items for systematic review and meta-analysis protocols (PRISMA-P) 2015 statement. Syst Rev. (2015) 4:1. doi: 10.1186/2046-4053-4-1, PMID: 25554246 PMC4320440

[39] Wang W Gong QL Zeng A Li MH Zhao Q Ni HB. Prevalence of cryptosporidium in pigs in China: a systematic review and meta-analysis. *Trans bound Emerg* Dis. (2021) 68:1400–13. doi: 10.1111/tbed.13806 32815651

[40] Ran X Cheng J Wang M Chen X Wang H Ge Y . Brucellosis seroprevalence in dairy cattle in China during 2008-2018: a systematic review and meta-analysis. Acta Trop. (2019) 189:117–23. doi: 10.1016/j.actatropica.2018.10.002, PMID: 30308207

[41] Barendregt JJ Doi SA Lee YY Norman RE Vos T. Meta-analysis of prevalence. J Epidemiol Community Health. (2013) 67:974–8. doi: 10.1136/jech-2013-203104, PMID: 23963506

[42] Assefa A Bihon A. Bovine cysticercosis in Ethiopia: a systematic review and meta-analysis of prevalence from abattoir-based surveys. Prev Vet Med. (2019) 169:104707. doi: 10.1016/j.prevetmed.2019.104707, PMID: 31311641

[43] Gong QL Li D Diao NC Liu Y Li BY Tian T . Mink Aleutian disease seroprevalence in China during 1981–2017: a systematic review and meta-analysis. Microb Pathog. (2020) 139:103908. doi: 10.1016/j.micpath.2019.103908, PMID: 31830583

[44] Shamseer L Moher D Clarke M Ghersi D Liberati A Petticrew M . Preferred reporting items for systematic review and meta-analysis protocols (PRISMA-P) 2015: elaboration and explanation. BMJ. (2015) 349:g7647. doi: 10.1136/bmj.g7647, PMID: 25555855

[45] Japanese encephalitis surveillance and immunization--Asia and the Western Pacific, 2012. *MMWR Morb Mortal Wkly* Rep. (2013) 62:658–62. PMID: 23965828 PMC4604796

[46] Dhanda V Thenmozhi V Kumar NP Hiriyan J Arunachalam N Balasubramanian A . Virus isolation from wild-caught mosquitoes during a Japanese encephalitis outbreak in Kerala in 1996. Indian J Med Res. (1997) 106:4–6. PMID: 9248207

[47] Schuh AJ Li L Tesh RB Innis BL Barrett AD. Genetic characterization of early isolates of Japanese encephalitis virus: genotype II has been circulating since at least 1951. J Gen Virol. (2010) 91:95–102. doi: 10.1099/vir.0.013631-0, PMID: 19776238 PMC2885061

[48] Williams DT Wang LF Daniels PW Mackenzie JS. Molecular characterization of the first Australian isolate of Japanese encephalitis virus, the Fu strain. J Gen Virol. (2000) 81:2471–80. doi: 10.1099/0022-1317-81-10-2471, PMID: 10993935

[49] Nabeshima T Loan HTK Inoue S Sumiyoshi M Haruta Y Nga PT . Evidence of frequent introductions of Japanese encephalitis virus from South-East Asia and continental East Asia to Japan. J Gen Virol. (2009) 90:827–32. doi: 10.1099/vir.0.007617-0, PMID: 19264633

[50] Sikazwe C Neave MJ Michie A Mileto P Wang J Cooper N . Molecular detection and characterization of the first Japanese encephalitis virus belonging to genotype IV acquired in Australia. PLoS Negl Trop Dis. (2022) 16:e0010754. doi: 10.1371/journal.pntd.0010754, PMID: 36409739 PMC9721490

[51] Sewgobind S Johnson N Mansfield KL. Jmm profile: Japanese encephalitis virus: an emerging threat. J Med Microbiol. (2022) 71. doi: 10.1099/jmm.0.001620, PMID: 36748429

[52] Zhao X Cao MQ Feng HH Fan H Chen F Feng Z . Japanese encephalitis risk and contextual risk factors in Southwest China: a Bayesian hierarchical spatial and spatiotemporal analysis. Int J Environ Res Public Health. (2014) 11:4201–17. doi: 10.3390/ijerph110404201, PMID: 24739769 PMC4024990

[53] Li X Gao X Ren Z Cao Y Wang J Liang G. A spatial and temporal analysis of Japanese encephalitis in Mainland China, 1963–1975: a period without Japanese encephalitis vaccination. PLoS One. (2014) 9:e99183. doi: 10.1371/journal.pone.0099183, PMID: 24911168 PMC4049618

[54] Zheng Y Li M Wang H Liang G. Japanese encephalitis and Japanese encephalitis virus in Mainland China. Rev Med Virol. (2012) 22:301–22. doi: 10.1002/rmv.1710, PMID: 22407526

[55] Liang G Li X Gao X Fu S Wang H Li M . Arboviruses and their related infections in China: a comprehensive field and laboratory investigation over the last 3 decades. Rev Med Virol. (2018) 28:1–21. doi: 10.1002/rmv.1959, PMID: 29210509

[56] Wang J Ogden NH Zhu H. The impact of weather conditions on Culex pipiens and *Culex restuans* (Diptera: Culicidae) abundance: a case study in Peel region. J Med Entomol. (2011) 48:468–75. doi: 10.1603/me10117, PMID: 21485391

[57] Wang L Hu W Soares Magalhaes RJ Bi P Ding F Sun H . The role of environmental factors in the spatial distribution of Japanese encephalitis in Mainland China. Environ Int. (2014) 73:1–9. doi: 10.1016/j.envint.2014.07.004, PMID: 25072160

[58] Aditi S Shailendra K Saxena Srivastava AK Asha M. Japanese encephalitis: a persistent threat. Proc Natl Acad Sci India Sect B Biol Sci. (2012) 82:55–68.

[59] Li RF Zhao XH Tian Y Shi YJ Gu XY Wang S. Different responses of Japanese encephalitis to weather variables among eight climate subtypes in Gansu, China, 2005-2019. BMC Infect Dis. (2023) 23:114. doi: 10.1186/s12879-023-08074-6, PMID: 36823521 PMC9951518

[60] Li XH Wei CH Dai AL Chen SY Yang XY. Epidemiological investigation of epidemic encephalitis B in pigs in Longyan city, China in Chinese. Heilongjiang Animal Sci Vet Med. (2017) 2:119–22. doi: 10.13881/j.cnki.hljxmsy.2017.0316

[61] Sun Y Ding H Zhao F Yan Q Li Y Niu X . Genomic characteristics and E protein bioinformatics analysis of JEV isolates from South China from 2011 to 2018. Vaccines (Basel). 10:1303. doi: 10.3390/vaccines10081303, PMID: 36016192 PMC9412759

[62] Beck C Lowenski S Durand B Bahuon C Zientara S Lecollinet S. Improved reliability of serological tools for the diagnosis of West Nile fever in horses within Europe. PLoS Negl Trop Dis. (2017) 11:e0005936. doi: 10.1371/journal.pntd.0005936, PMID: 28915240 PMC5617233

[63] Shao N Li F Nie K Fu SH Zhang WJ . TaqMan real-time RT-PCR assay for detecting and differentiating Japanese encephalitis virus. Biomed Environ Sci. (2018) 31:208–14. doi: 10.3967/bes2018.026 29673443

[64] Santhosh SR Parida MM Dash PK Pateriya A Pattnaik B Pradhan HK . Development and evaluation of SYBR green I-based one-step real-time RT-PCR assay for detection and quantitation of Japanese encephalitis virus. J Virol Methods. (2007) 143:73–80. doi: 10.1016/j.jviromet.2007.02.011, PMID: 17403544

[65] Wu X Lin H Chen S Xiao L Yang M An W . Development and application of a reverse transcriptase droplet digital PCR (RT-ddPCR) for sensitive and rapid detection of Japanese encephalitis virus. J Virol Methods. (2017) 248:166–71. doi: 10.1016/j.jviromet.2017.06.015, PMID: 28673857

[66] Cui Y. Development of Nanobody and construction of immunoassay for staphylococcal enterotoxin (in Chinese). Northwest A&F Univ. (2024). doi: 10.27409/d.cnki.gxbnu.2024.002195

[67] Sun SY Wu JJ Yang Z. Progress in serological detection of hepatitis B. Modern Med Health Res Electronic J. (2023) 7:134–7.

[68] Niu YH Yang K Wang FM Shen HQ Zhao BH. Research advances and application of the polymerase chain reaction (PCR) in shrimp virus inspection. Hebei Fisheries. (2009) 1:14–24.

[69] Liu ZL Liu ZY Li ZJ Guo L. Research progress of bovine rotavirus. Graziery Vet Sci. (2019) 20:1–3.

[70] Mei L Wu P Ye J Gao G Shao L Huang S . Development and application of an antigen capture ELISA assay for diagnosis of Japanese encephalitis virus in swine, human and mosquito. Virol J. (2012) 9:4. doi: 10.1186/1743-422X-9-4, PMID: 22221768 PMC3275457

[71] Li Y Hou L Ye J Liu X Dan H Jin M . Development of a convenient immunochromatographic strip for the diagnosis of infection with Japanese encephalitis virus in swine. J Virol Methods. (2010) 168:51–6. doi: 10.1016/j.jviromet.2010.04.015, PMID: 20433870

[72] Liu Y Gong QL Nie LB Wang Q Ge GY Li DL . Prevalence of porcine circovirus 2 throughout China in 2015–2019: a systematic review and meta-analysis. Microb Pathog. (2020) 149:104490. doi: 10.1016/j.micpath.2020.104490, PMID: 32956791

[73] Zheng B Wang X Liu Y Li Y Long S Gu C . Japanese encephalitis virus infection induces inflammation of swine testis through RIG-I-NF-kB signaling pathway. Vet Microbiol. (2019) 238:108430. doi: 10.1016/j.vetmic.2019.108430, PMID: 31648727

[74] Liu H Liu ZJ Jing J Ren JQ Liu YY Guo HH . Reverse transcription loop-mediated isothermal amplification for rapid detection of Japanese encephalitis virus in swine and mosquitoes. Vector Borne Zoonotic Dis. (2012) 12:1042–52. doi: 10.1089/vbz.2012.0991, PMID: 23176446 PMC3525893

[75] Zeng Q Liu J Li Z Zhang Y Zu S Ding X . Japanese encephalitis virus NS4B inhibits interferon beta production by targeting TLR3 and TRIF. Vet Microbiol. (2023) 284:109849. doi: 10.1016/j.vetmic.2023.109849, PMID: 37597377

[76] Pierson TC Diamond MS. The continued threat of emerging flaviviruses. Nat Microbiol. (2020) 5:796–812. doi: 10.1038/s41564-020-0714-0, PMID: 32367055 PMC7696730

[77] Li X Li J Wu G Wang M Jing Z. Detection of Japanese encephalitis by metagenomic next-generation sequencing of cerebrospinal fluid: a case report and literature review. Front Cell Neurosci. (2022) 16:856512. doi: 10.3389/fncel.2022.856512, PMID: 35250491 PMC8892252

[78] Sharma KB Vrati S Kalia M. Pathobiology of Japanese encephalitis virus infection. Mol Asp Med. (2021) 81:100994. doi: 10.1016/j.mam.2021.100994, PMID: 34274157

[79] National Health Commission of the people′s republic of China. Immunization schedules and instructions for vaccines of the national immunization program (2021 version). Chinese J Viral Dis. (2021) 4:241–5. doi: 10.16505/j.2095-0136.2021.0021

[80] Global Advisory Committee on vaccine safety, 9-10 June 2005. Wkly Epidemiol Rec. (2005) 80:242–7. PMID: 16047931

[81] Tu T Xu KQ Xu L Gao Y Zhou Y He YM . Association between meteorological factors and the prevalence dynamics of Japanese encephalitis. PLoS One. (2021) 16:e0247980. doi: 10.1371/journal.pone.0247980, PMID: 33657174 PMC7928514

[82] Li LH Li Y Bi YH. Mosquito and pig with Japanese encephalitis (in Chinese). Swine Ind Sci. (2008) 6:34–6.

[83] Sakamoto R Tanimoto T Takahashi K Hamaki T Kusumi E Crump A. Flourishing Japanese encephalitis, associated with global warming and urbanisation in Asia, demands widespread integrated vaccination programmes. Ann Glob Health. (2019) 85:111. doi: 10.5334/aogh.2580, PMID: 31373473 PMC6676921

[84] Walsh MG Pattanaik A Vyas N Saxena D Webb C Sawleshwarkar S . High-risk landscapes of Japanese encephalitis virus outbreaks in India converge on wetlands, rain-fed agriculture, wild Ardeidae, and domestic pigs and chickens. Int J Epidemiol. (2022) 51:1408–18. doi: 10.1093/ije/dyac050, PMID: 35355081 PMC9557850

[85] Ladreyt H Durand B Dussart P Chevalier V. How central is the domestic pig in the epidemiological cycle of Japanese encephalitis virus? A review of scientific evidence and implications for disease control. Viruses. (2019) 11:949. doi: 10.3390/v11100949, PMID: 31618959 PMC6832429

[86] Tang Q Deng Z Tan S Song G Zhang H Ge L. Prevalence and genetic characteristics of Japanese encephalitis virus among mosquitoes and pigs in Hunan Province, China from 2019 to 2021. J Microbiol Biotechnol. (2022) 32:1120–5. doi: 10.4014/jmb.2207.07068, PMID: 36116917 PMC9628968

