# Supplementary material for: Prevalence of Japanese encephalitis in pigs in Mainland China during 2000–2024: a systemic review and meta-analysis
Source: Front Vet Sci. 2025 Feb 7;12:1534114. doi: 10.3389/fvets.2025.1534114 (PMC11842376; doi:10.3389/fvets.2025.1534114)

**Table S1.** PRISMA Checklist item.

| **Section/topic** | **#** | **Checklist item** |
| --- | --- | --- |
| **TITLE** |  |  |
| Title | 1 | Prevalence of Epidemic encephalitis B in Pigs in mainland China during 2000-2024: A systemic review and meta-analysis |
| **ABSTRACT** |  |  |
| Structured summary | 2 | Provide a structured summary including, as applicable: background; objectives; data sources; study eligibility criteria, participants, and interventions; study appraisal and synthesis methods; results; limitations; conclusions and implications of key findings; systematic review registration number. |
| **INTRODUCTION** |  |  |
| Rationale | 3 | Describe the rationale for the review in the context of what is already known. |
| Objectives | 4 | Provide an explicit statement of questions being addressed with reference to participants, interventions, comparisons, outcomes, and study design (PICOS). |
| **METHODS** |  |  |
| Protocol and registration | 5 | Indicate if a review protocol exists, if and where it can be accessed (e.g., Web address), and, if available, provide registration information including registration number. |
| Eligibility criteria | 6 | Specify study characteristics (e.g., PICOS, length of follow-up) and report characteristics (e.g., years considered, language, publication status) used as criteria for eligibility, giving rationale. |
| Information sources | 7 | Describe all information sources (e.g., databases with dates of coverage, contact with study authors to identify additional studies) in the search and date last searched. |
| Search | 8 | Present full electronic search strategy for at least one database, including any limits used, such that it could be repeated. |
| Study selection | 9 | State the process for selecting studies (i.e., screening, eligibility, included in systematic review, and, if applicable, included in the meta-analysis). |
| Data collection process | 10 | Describe method of data extraction from reports (e.g., piloted forms, independently, in duplicate) and any processes for obtaining and confirming data from investigators. |
| Data items | 11 | List and define all variables for which data were sought (e.g., PICOS, funding sources) and any assumptions and simplifications made. |
| Risk of bias in individual studies | 12 | Describe methods used for assessing risk of bias of individual studies (including specification of whether this was done at the study or outcome level), and how this information is to be used in any data synthesis. |
| Summary measures | 13 | State the principal summary measures (e.g., risk ratio, difference in means). |
| Synthesis of results | 14 | Describe the methods of handling data and combining results of studies, if done, including measures of consistency (e.g., I^2^) for each meta-analysis. |
| Risk of bias across studies | 15 | Specify any assessment of risk of bias that may affect the cumulative evidence (e.g., publication bias, selective reporting within studies). |
| Additional analyses | 16 | Describe methods of additional analyses (e.g., sensitivity or subgroup analyses, meta-regression), if done, indicating which were pre-specified. |
| **RESULTS** |  |  |
| Study selection | 17 | Give numbers of studies screened, assessed for eligibility, and included in the review, with reasons for exclusions at each stage, ideally with a flow diagram. |
| Study characteristics | 18 | For each study, present characteristics for which data were extracted (e.g., study size, PICOS, follow-up period) and provide the citations. |
| Risk of bias within studies | 19 | Present data on risk of bias of each study and, if available, any outcome level assessment (see item 12). |
| Results of individual studies | 20 | For all outcomes considered (benefits or harms), present, for each study: (a) simple summary data for each intervention group (b) effect estimates and confidence intervals, ideally with a forest plot. |
| Synthesis of results | 21 | Present results of each meta-analysis done, including confidence intervals and measures of consistency. |
| Risk of bias across studies | 22 | Present results of any assessment of risk of bias across studies (see Item 15). |
| Additional analysis | 23 | Give results of additional analyses, if done (e.g., sensitivity or subgroup analyses, meta-regression [see Item 16]). |
| **DISCUSSION** |  |  |
| Summary of evidence | 24 | Summarize the main findings including the strength of evidence for each main outcome; consider their relevance to key groups (e.g., healthcare providers, users, and policy makers). |
| Limitations | 25 | Discuss limitations at study and outcome level (e.g., risk of bias), and at review-level (e.g., incomplete retrieval of identified research, reporting bias). |
| Conclusions | 26 | Provide a general interpretation of the results in the context of other evidence, and implications for future research. |
| **FUNDING** |  |  |
| Funding | 27 | Describe sources of funding for the systematic review and other support (e.g., supply of data); role of founders for the systematic review. |

*From:* Moher D, Liberati A, Tetzlaff J, Altman DG, The PRISMA Group (2009). Preferred Reporting Items for Systematic Reviews and Meta-Analyses: The PRISMA Statement. PLoS Med 6(6): e1000097. doi:10.1371/journal.pmed1000097

For more information, visit: **www.prisma-statement.org**.

**Table S2.** The search formulas for the six databases

| PubMed | ((((((("Encephalitis, Japanese"[Mesh]) OR (Encephalitis, Japanese B)) OR (Viral Encephalitis, Japanese B)) OR (Japanese B Viral Encephalitis)) OR (Japanese B Encephalitis)) OR (Japanese Encephalitis)) AND ((((((((("Swine"[Mesh]) OR (Pig)) OR (Pigs)) OR (Sus scrofa domesticus)) OR (Sus scrofa domestica)) OR (Domestic Pig)) OR (Pig, Domestic)) OR (Domestic Pigs)) OR (Pigs, Domestic))) AND (((((("China"[Mesh]) OR (People's Republic of China)) OR (Mainland China)) OR (Sinkiang)) OR (Inner Mongolia)) OR (Manchuria)) |
| --- | --- |
| ScienceDirect | prevalence, Swine Japanese B Encephalitis, China |
| Web of Science | Japanese B Encephalitis (Topic) and Swine (Topic) and prevalence (Topic) |
| CNKI | Japanese B Encephalitis + Epidemic encephalitis B + Infection rate + prevalence + prevalence rate + positive rate + epidemiological investigation + application in Chinese |
| Wan Fang | Japanese B Encephalitis + Epidemic encephalitis B + Infection rate + prevalence + prevalence rate + positive rate + epidemiological investigation + application in Chinese |
| VIP | Japanese B Encephalitis + Epidemic encephalitis B + Infection rate + prevalence + prevalence rate + positive rate + epidemiological investigation + application in Chinese |

**Table S3.** The code in R for meta-analysis.

| No transformation (PRAW) | rate<-transform[m1, r= event/n];  shapiro.test(rate$r) |
| --- | --- |
| Logarithmic conversion (PLN) | rate<-transform [m1, log=log(event/n)];  shapiro.test(rate$log) |
| Logit transformation (PLOGIT) | rate<-transform{m1, logit=log[(event/n)/(1-event/n)]};  shapiro.test(rate$logit) |
| Arcsine transformation (PAS) | rate<-transform{m1, arcsin.size=asin[sqrt(event/(n+1))]};  shapiro.test(rate$arcsin) |
| Double-arcsine transformation (PFT) | rate<-transform{m1,darcsin=0.5*[asin(sqrt(event/(n+1)))+asin((sqrt(event+1)/(n+1)))]};  shapiro.test(rate$darcsin) |
| Forest plots | forest [meta1, xlim=c(-0.2, 1)] |
| Funnel chart | funnel (meta1) |
| Egger's test | metabias (meta1, method="linreg") |
| The sensitivity analysis | metainf (meta1, pooled = "random") forest (metainf (meta1, pooled = "random"), xlim=c(0, 0.6)) |
| Subgroup analysis | meta1<-metaprop(event, n, study, data=rate, sm="PFT", incr=0.5, allincr=TRUE, addincr=FALSE, title="", byvar= subgroup title, print.byvar=TRUE) |
| Meta-regression analysis | metareg (meta1, ~covariate title) |

**Table S4.** Included studies and quality scores**.**

|  | **Reference ID** | **No. tested** | **No. positive** | **Prevalence** | **Random sampling or not** | **Sampled method detailly or not** | **Sample time clearly or not** | **Detection method clearly or not** | **Four or more risk factors or not** | **Score** |
| --- | --- | --- | --- | --- | --- | --- | --- | --- | --- | --- |
| 1 | Fan et al. (2014) | 1056 | 455 | 0.431 | N | N | Y | Y | Y | 3 |
| 2 | Yao et al. (2022) | 1523 | 1199 | 0.787 | N | N | N | Y | Y | 2 |
| 3 | Huang et al. (2012) | 4282 | 2227 | 0.520 | N | N | Y | Y | Y | 3 |
| 4 | Hua and Li (2012) | 2906 | 1239 | 0.426 | N | N | N | Y | Y | 2 |
| 5 | Ma et al. (2020) | 465 | 445 | 0.957 | N | N | Y | Y | Y | 3 |
| 6 | Zhao et al. (2023) | 3105 | 60 | 0.019 | N | N | Y | Y | Y | 3 |
| 7 | Li et al. (2018) | 135 | 93 | 0.689 | N | N | Y | Y | Y | 3 |
| 8 | Yang et al. (2013) | 135 | 67 | 0.496 | N | N | Y | Y | Y | 3 |
| 9 | Zhou. (2011) | 274 | 12 | 0.044 | N | N | N | Y | Y | 2 |
| 10 | Tang et al. (2022) | 3026 | 583 | 0.193 | N | N | Y | Y | Y | 3 |
| 11 | Jiang and Liu. (2007) | 233 | 132 | 0.567 | Y | Y | Y | N | Y | 4 |
| 12 | Sun et al. (2012) | 866 | 88 | 0.102 | Y | Y | N | Y | Y | 4 |
| 13 | Liu et al. (2006) | 86 | 21 | 0.244 | Y | N | Y | N | N | 2 |
| 14 | Chen et al. (2000) | 149 | 41 | 0.107 | Y | N | Y | N | N | 2 |
| 15 | Jin et al. (2008) | 172 | 13 | 0.076 | N | N | Y | Y | Y | 3 |
| 16 | Cui. (2018) | 801 | 384 | 0.479 | N | N | Y | Y | Y | 3 |
| 17 | Liu et al. (2007) | 592 | 194 | 0.328 | N | N | Y | Y | Y | 3 |
| 18 | Yang et al. (2008) | 2292 | 1105 | 0.482 | N | N | Y | Y | Y | 3 |
| 19 | Zhang et al. (2017) | 454 | 23 | 0.051 | N | N | N | Y | Y | 2 |
| 20 | Nie et al. (2022) | 185 | 12 | 0.065 | N | N | Y | Y | Y | 3 |
| 21 | Guo et al. (2019) | 167 | 11 | 0.066 | N | N | Y | Y | Y | 3 |
| 22 | Chai et al. (2018) | 22343 | 8807 | 0.394 | N | N | Y | N | Y | 2 |
| 23 | Liu et al. (2013) | 108 | 20 | 0.185 | N | N | Y | Y | Y | 3 |
| 24 | Ceng and Chen. (2011) | 592 | 355 | 0.600 | N | N | Y | Y | Y | 3 |
| 25 | Qin and He. (2011) | 2597 | 1575 | 0.607 | N | N | Y | Y | Y | 3 |
| 26 | Zhang and Lu. (2011) | 2161 | 592 | 0.274 | N | N | Y | Y | Y | 3 |
| 27 | Chen and Wei. (2010) | 23 | 1 | 0.043 | N | Y | Y | Y | Y | 4 |
| 28 | Li et al. (2009) | 152 | 33 | 0.217 | N | N | Y | Y | Y | 3 |
| 29 | Wu et al. (2024) | 486 | 124 | 0.255 | N | Y | Y | Y | Y | 4 |
| 30 | Li et al. (2010) | 1676 | 923 | 0.551 | N | Y | Y | Y | Y | 4 |
| 31 | Jiang et al. (2010) | 216 | 144 | 0.667 | N | N | Y | Y | Y | 3 |

Y*: Yes; N*: No.

**References**

Fan, Y.; Cao, Y.B.; Chen, P.Y. Epidemiologic Survey of Wild-Virus Infection of Porcine B Encephalitis in Different Ecological Regions of China in 2014(in Chinese). In Proceedings of the Proceedings of the 2014 Annual Academic Conference of the Chinese Society of Animal Husbandry and Veterinary Medicine(in Chinese), 2014; p. 1.

Yao, Y.L.; Song, J.G.; Kang, W.B.; Zhou, F.; Dou, L.; Gao, J.J.; Pan, Y.H. Prevalence Situation Investigation on Swine Japanese Encephalitis in Partial Small and Medium-Size Farms of Gansu Province. *Progress in Veterinary Medicine* **2022**, *43*, 126-129, doi:10.16437/j.cnki.1007-5038.2022.09.004.

Huang, Y.H.; Yin., Z.X.; Xie, G.H.; Yin, S.H.; Zhang, X.P. Epidemiological Investigation of Porcine Japanese Encephalitis in Dongguan City of Guangdong Province(in Chinese). *Progress in Veterinary Medicine* **2012**, *33*, 126-128, doi:10.16437/j.cnki.1007-5038.2012.07.030.

Hua, Y.; Li., J.Z. Serological Investigation on Swine Japanese Encephalitis in Guizhou(in Chinese). *Guizhou Agricultural Sciences* **2012**, *40*, 102-103.

Ma, S.J.; Zhong., X.S.; Li, D.L.; Xiong, Y.Q.; Zheng,X.Y.; Jiang, L.N.; Chen, S.W.; Chen, W. . Japanese Encephalitis Virus Infection in Domestic Swine in Some Areas of Southern China:A Serological Survey(in Chinese). *Chinese Journal of Public Health* **2020**, *36*, 375-377.

Zhao, G.; Gao, Y.; Shi, N.; Zhang, S.; Xiao, P.; Zhang, J.; Xie, C.; Ha, Z.; Feng, S.; Li, C.; et al. Molecular Detection and Genetic Characterization of Japanese Encephalitis Virus in Animals from 11 Provinces in China(in Chinese). *Viruses* **2023**, *15*, doi:10.3390/v15030625.

Li, X.H.; Wei., C.H.; Dai, A.L.; Chen, S.Y.; Yang, X.Y. Epidemiologic Investigation of Epidemic Encephalitis B in Pigs in Longyan City, China. In Proceedings of the The Sixth Symposium of the Veterinary Public Health Branch of the Chinese Society of Animal Husbandry and Veterinary Medicine(in Chinese), 2018; p. 1.

Yang, M.Y.; Yang, P.C.; Xiao, J.; Wen, Z. Serologic Survey on the Prevalence of Swine Encephalitis B in Wuding County, China(in Chinese). *Modern Animal Husbandry Science & Technology* **2013**, 76.

Zhou, Q.Y. Epidemiological Survey of Porcine Reproductive Disorders Syndrome in Mengzi County, Yunnan Province, China(in Chinese). *Animals Breeding and Feed* **2011**, 7-10, doi:10.13300/j.cnki.cn42-1648/s.2011.02.005.

Tang, Q.; Deng, Z.; Tan, S.; Song, G.; Zhang, H.; Ge, L. Prevalence and Genetic Characteristics of Japanese Encephalitis Virus among Mosquitoes and Pigs in Hunan Province, China from 2019 to 2021(in Chinese). *J Microbiol Biotechnol* **2022**, *32*, 1120-1125, doi:10.4014/jmb.2207.07068.

Wang, D.M.; Liu., M.Z. Report on the Serologic Survey of 8 Epidemic Diseases in 6 Pig Farms in Gansu Province(in Chinese). *Shanghai Journal of Animal Husbandry and Veterinary Medicine* **2007**, 56-57.

Sun, G.L.; Sun, G.; Guo, S.L.; Yang, B.; Xu, B.; Dong, F. Epidemiologic Survey of Encephalitis B in Pig Breeding Farms in Heilongjiang Province(in Chinese). *Heilongjiang Animal Science and Veterinary Medicine* **2002**, 29-30, doi:10.13881/j.cnki.hljxmsy.2002.03.020.

Liu, B.S.; Wang, Q.Q.; Li, Y.B.; Guo, J.X.; Liu, J.S.; Zhou, X.W. Epidemiologic Investigation of Swine Encephalitis B Disease in Honghe Prefecture(in Chinese). *Yunnan Journal of Animal Science and Veterinary Medicine* **2006**, 17-18.

Chen, Z.M.; Tang, H.P.; Zhang, C.Y.; Zeng, Z.H. Serological Investigation of Pseudorabies, Microbial Virus Disease, Encephalitis B and Brucellosis in Pigs in Zhangzhou City, China(in Chinese). *Fujian Zhangzhou City Animal Husbandry and Veterinary Station* **2001**, *23*, 1-1.

Jin, X.J.; Han, L.; Ji, W.H.; Luo, F.B.; Liang, M.Z.; Gao, Y.L.; Guo, F.; Shen, G.N. Seroepidemiologic Survey of Porcine Encephalitis B in Beijing, China(in Chinese). *Chinese Journal of Veterinary Medicine* **2008**, *44*, 51-52, doi:10.3969/j.issn.0529-6005.2008.08.025.

Cui, G.H. Seroepidemiologic Survey of Swine Encephalitis B in Henan Province and Isolation and Characterization of the Local Strain of Encephalitis B Virus in Henan Province(in Chinese). Henan Agricultural University, 2009.

Liu, Z.Y.; Liu, C.F.; Cui, L.Y.; Wu, W.B.; Liu, D.Q. Surveillance of Swine Epidemic Encephalitis B Virus Infection in Kaijiang County, 2002-2006(in Chinese). *Chinese Preventive Medicine* **2008**, *9*, 138-139, doi:10.3969/j.issn.1009-6639.2008.02.016.

Yang, P.C.; Cao, X.P.; Gao, Z.S.; Zhang,L.F.; Wang,B. Serologic Survey on the Prevalence of Porcine Encephalitis B in Chuxiong Prefecture, Yunnan Province, China(in Chinese). *Progress in Veterinary Medicine* **2008**, *29*, 109-112, doi:10.3969/j.issn.1007-5038.2008.05.029.

Zhang, H.; Rehman, M.U.; Li, K.; Luo, H.; Lan, Y.; Nabi, F.; Zhang, L.; Iqbal, M.K.; Zhu, S.; Javed, M.T.; et al. Epidemiologic Survey of Japanese Encephalitis Virus Infection, Tibet, China, 2015. *Emerg Infect Dis* **2017**, *23*, 1023-1024, doi:10.3201/eid2306.152115.

Nie, M.C.; Zhou, Y.C.; Li, F.Q.; Deng, H.D.; Zhao, M.X.; Huang, Y.; Jiang, C.Y.; Sun, X.G.; Xu, Z.W.; Zhu, L. Epidemiological Investigation of Swine Japanese Encephalitis Virus Based on Rt-Raa Detection Method(Ss). *SCIENTIFIC REPORTS* **2022**, *12*, doi:10.1038/s41598-022-13604-4.

Guo, H.-c.; Ren, Z.-w.; Ding, M.-m.; Xiao, W.-j.; Peng, P.; He, B.; Feng, Y.; Liu, Y.; Li, X.-y.; Cai, J.-q.; et al. Serologic and Molecular Survey for Major Viral Pathogens in Grazing Hybrid Wild Boars in Northeast China. *Journal of Integrative Agriculture* **2019**, *18*, 2133-2140, doi:https://doi.org/10.1016/S2095-3119(19)62650-X.

Chai, C.; Wang, Q.; Cao, S.; Zhao, Q.; Wen, Y.; Huang, X.; Wen, X.; Yan, Q.; Ma, X.; Wu, R. Serological and Molecular Epidemiology of Japanese Encephalitis Virus Infections in Swine Herds in China, 2006-2012（Ss）. *J Vet Sci* **2018**, *19*, 151-155, doi:10.4142/jvs.2018.19.1.151.

Liu, H.; Lu, H.J.; Liu, Z.J.; Jing, J.; Ren, J.Q.; Liu, Y.Y.; Lu, F.; Jin, N.Y. Japanese Encephalitis Virus in Mosquitoes and Swine in Yunnan Province, China 2009-2010. *Vector Borne Zoonotic Dis* **2013**, *13*, 41-49, doi:10.1089/vbz.2012.1016.

Cheng, Z.T.; Chen., J.Y.; Yue, Y.; Zhou, B.J.; Wen, M.; Wang, K.g.; Long, H.; Li, J.D. The Seroepidemiological Survey of Porcine Epidemic Encephalitis B Inguizhou Province(in Chinese). *Chinese Journal of Zoonoses* **2011**, *27*, 1156-1158, doi:10.3969/j.issn.1002-2694.2011.12.022.

Qin, Y.B.; He, Y.; He, P.P.; Li, B.; Lu, B.X.; Zhao, W.; Liang, J.X.; Su, Q.L.; Yan, Z.B.; Li, C.T.; Liu, F.; Liang, B.Z.; Huang,W.J. Serological Epidemiology of Japanese Encephalitis Virus Infection to Swinein Guangxi(in Chinese). *Journal of Southern Agriculture* **2011**, *42*, 668-671, doi:10.3969/j.issn.2095-1191.2011.06.027.

Zhang, D.; Lu; H.J.; Jin, K.S.; Jin, N.Y.; Tan, L.; Liu, H.; Liu, Y.Y. Seroepidemiologic Survey and Analysis of Porcine Epidemic Encephalitis B in Northeast China(in Chinese). In Proceedings of the Heilongjiang Animal Science and Veterinary Medicine, 2010; p. 3.

Chen, H.Y.; Wei, Z.Y.; Zhang, H.Y.; Lü, X.L.; Zheng, L.L.; Cui, B.A.; Liu, J.; Zhu, Q.L.; Wang, Z.X. Use of a Multiplex Rt-Pcr Assay for Simultaneous Detection of the North American Genotype Porcine Reproductive and Respiratory Syndrome Virus, Swine Influenza Virus and Japanese Encephalitis Virus. *Agric Sci China* **2010**, *9*, 1050-1057, doi:10.1016/s1671-2927(09)60189-9.

Li, Y.T.; Zhu, Y.Y.; Jin, B.H. [Epidemiological Characteristics of Japanese Encephalitis in Shanghai]. *Zhongguo Yi Miao He Mian Yi* **2009**, *15*, 507-510, 561.

Wu, Y. Serologic Investigation and Analysis of Four Important Swine Diseases in Nanchong Area, Sichuan Province, China(in Chinese). Sichuan Agricultural University, 2009.

Li, M.N. Isolation and Characterization of Porcine Encephalitis B Virus in Guangxi and Seroepidemiologic Survey Studies(in Chinese). Guangxi University, 2010.

Jiang, Y.H. Survey on the Status of Encephalitis B Virus Infection in the Three Gorges Reservoir Area of China(in Chinese). Shandong University, 2010.

**Figure S1. Funnel plot with pseudo 95% confidence limits intervals for the examination of publication bias of region**


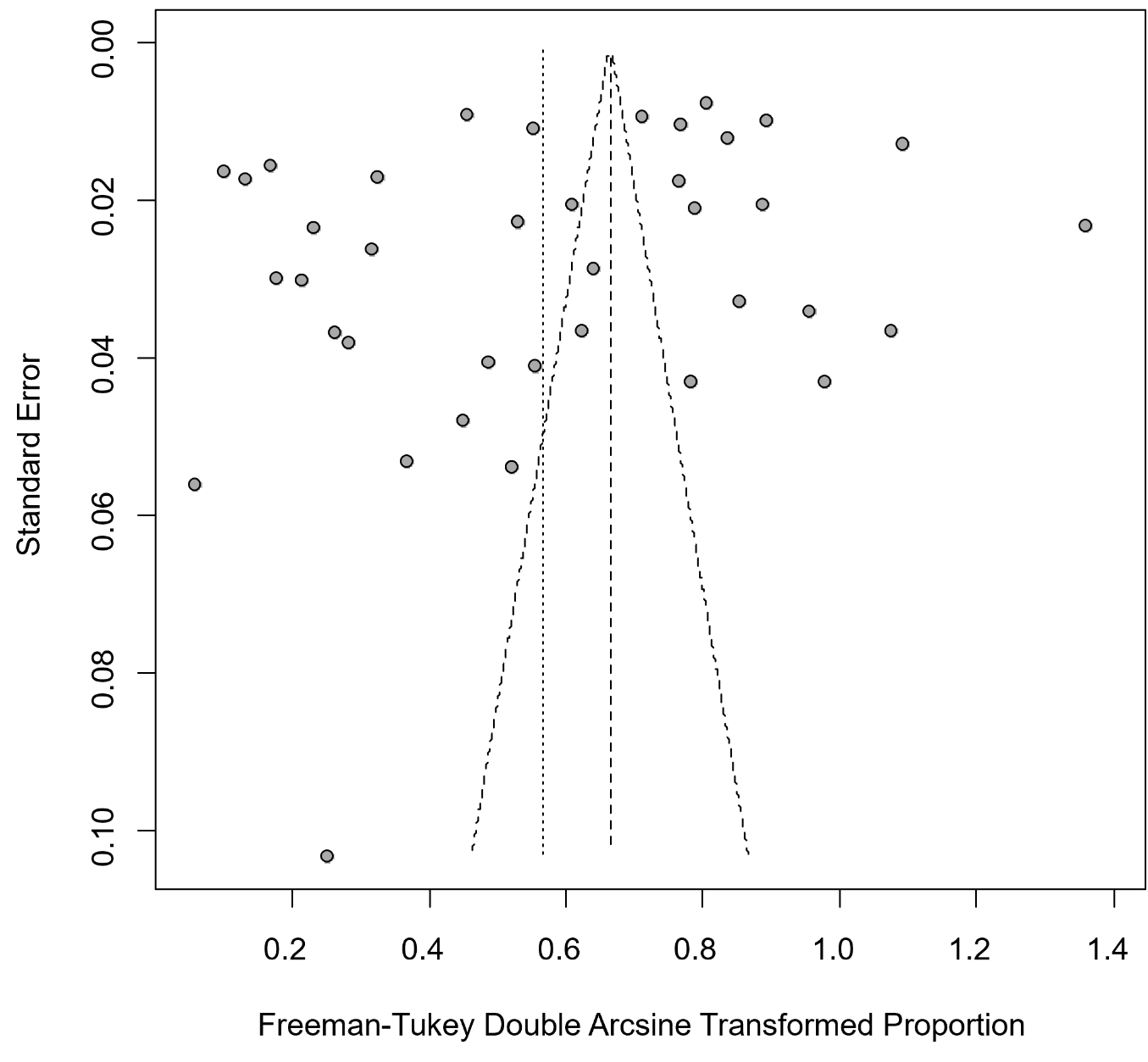


**Figure S2. Funnel plot with pseudo 95% confidence limits intervals for the examination of publication bias of sampling years**

**
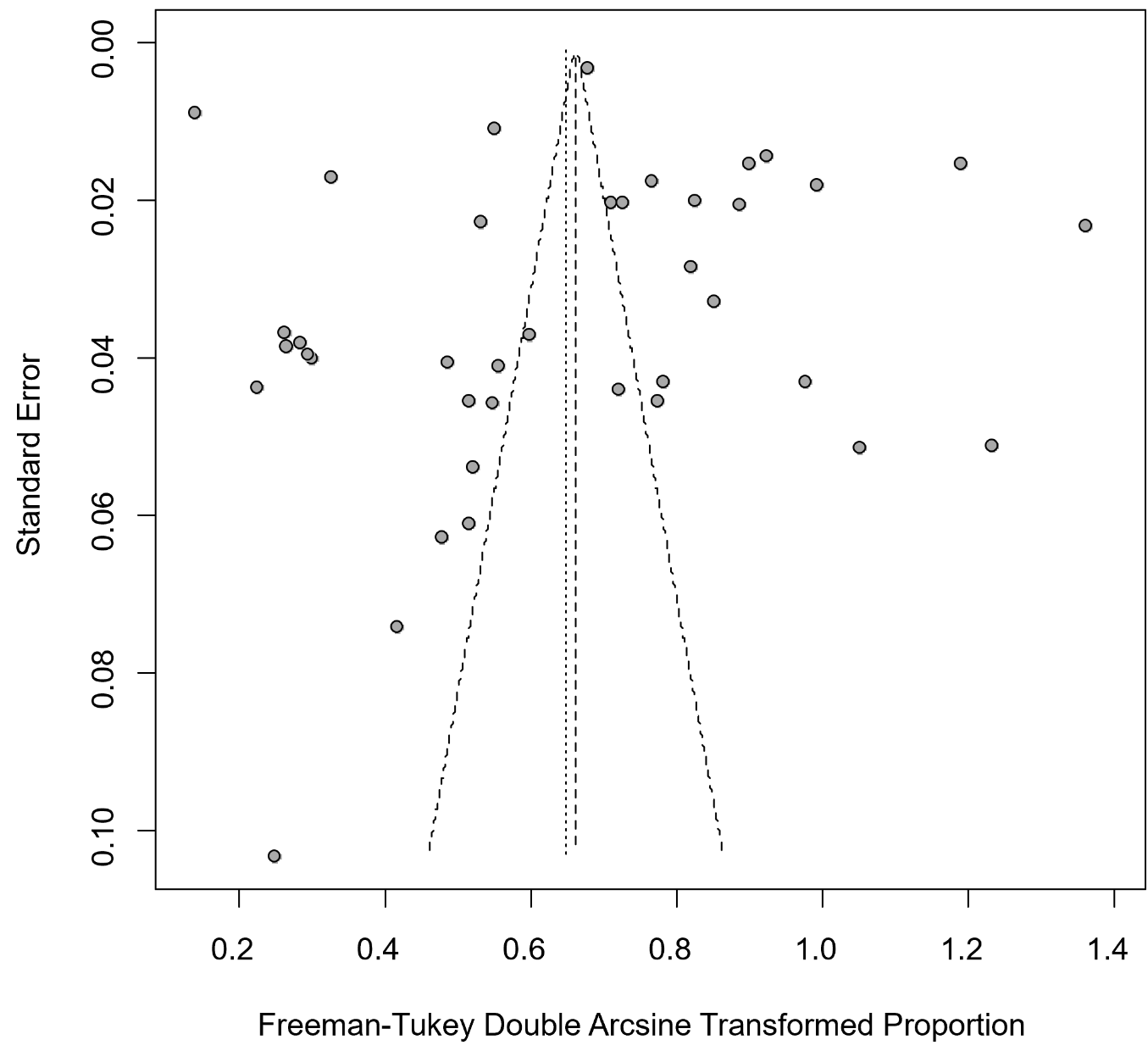
**

**Figure S3. Funnel plot with pseudo 95% confidence limits intervals for the examination of publication bias of sample**

**
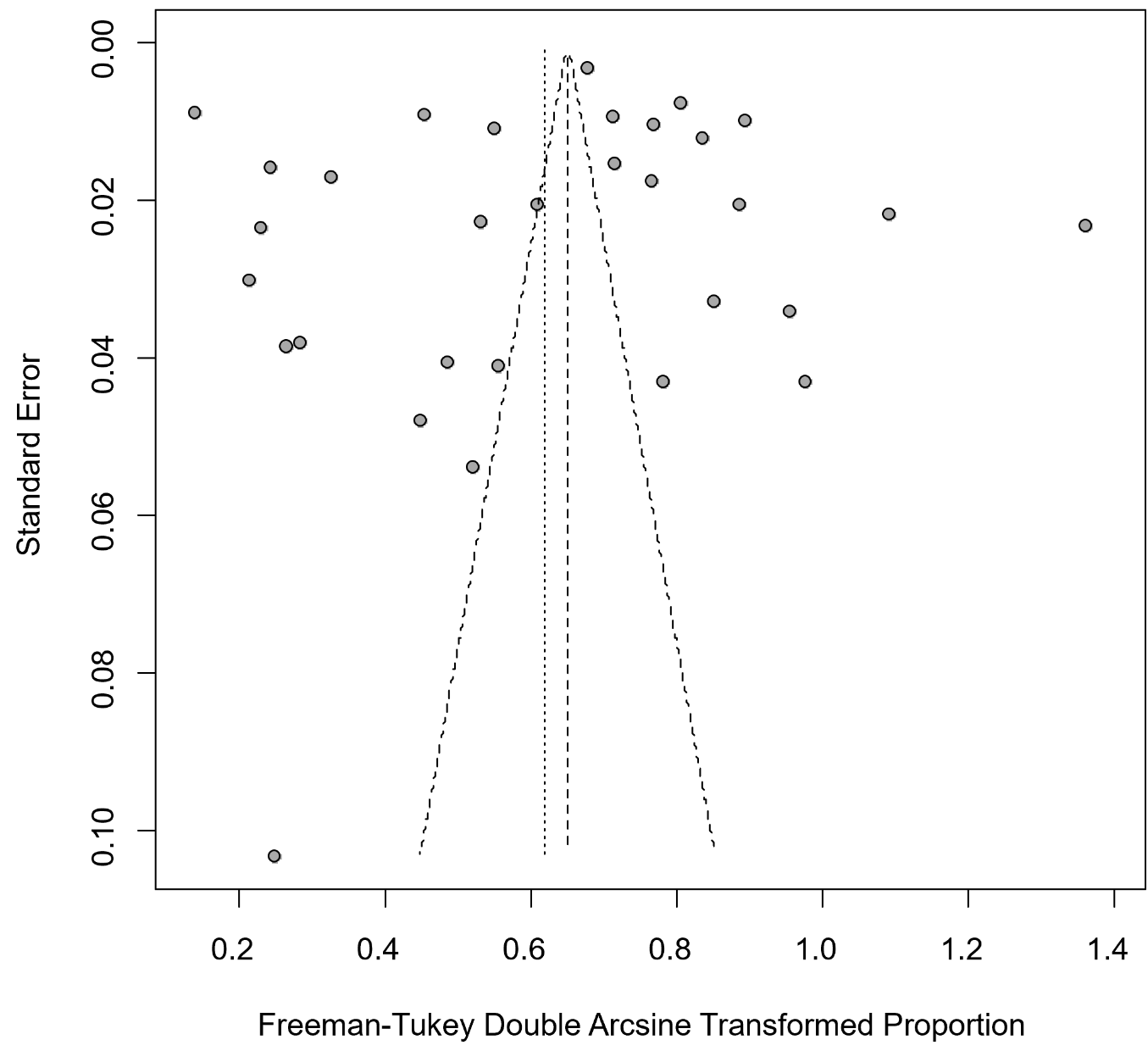
**

**Figure S4. Funnel plot with pseudo 95% confidence limits intervals for the examination of publication bias of detection method**


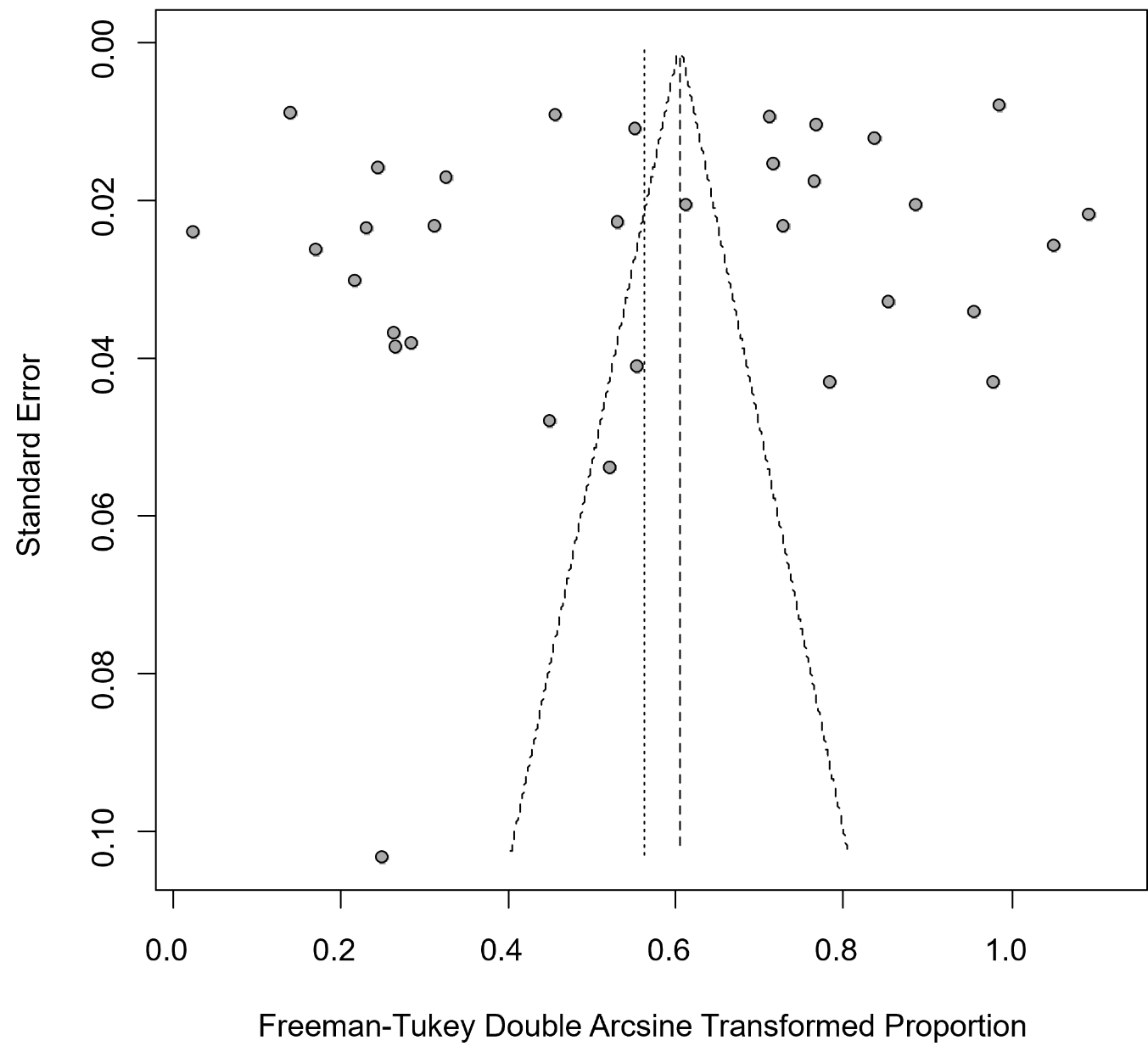


**Figure S5. Funnel plot with pseudo 95% confidence limits intervals for the examination of publication bias of** **breeding mode**


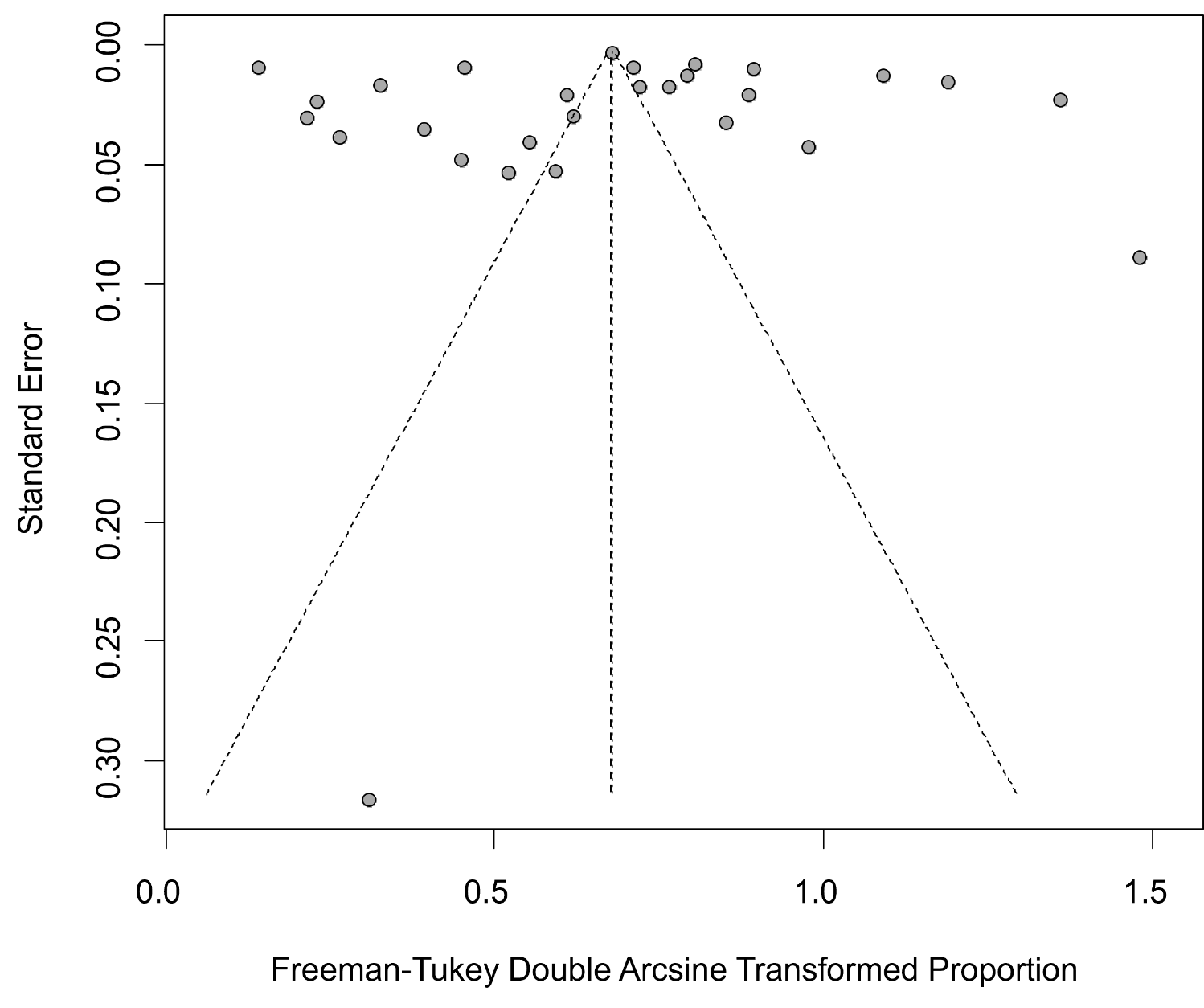


**Figure S6. Funnel plot with pseudo 95% confidence limits intervals for the examination of publication bias of season**


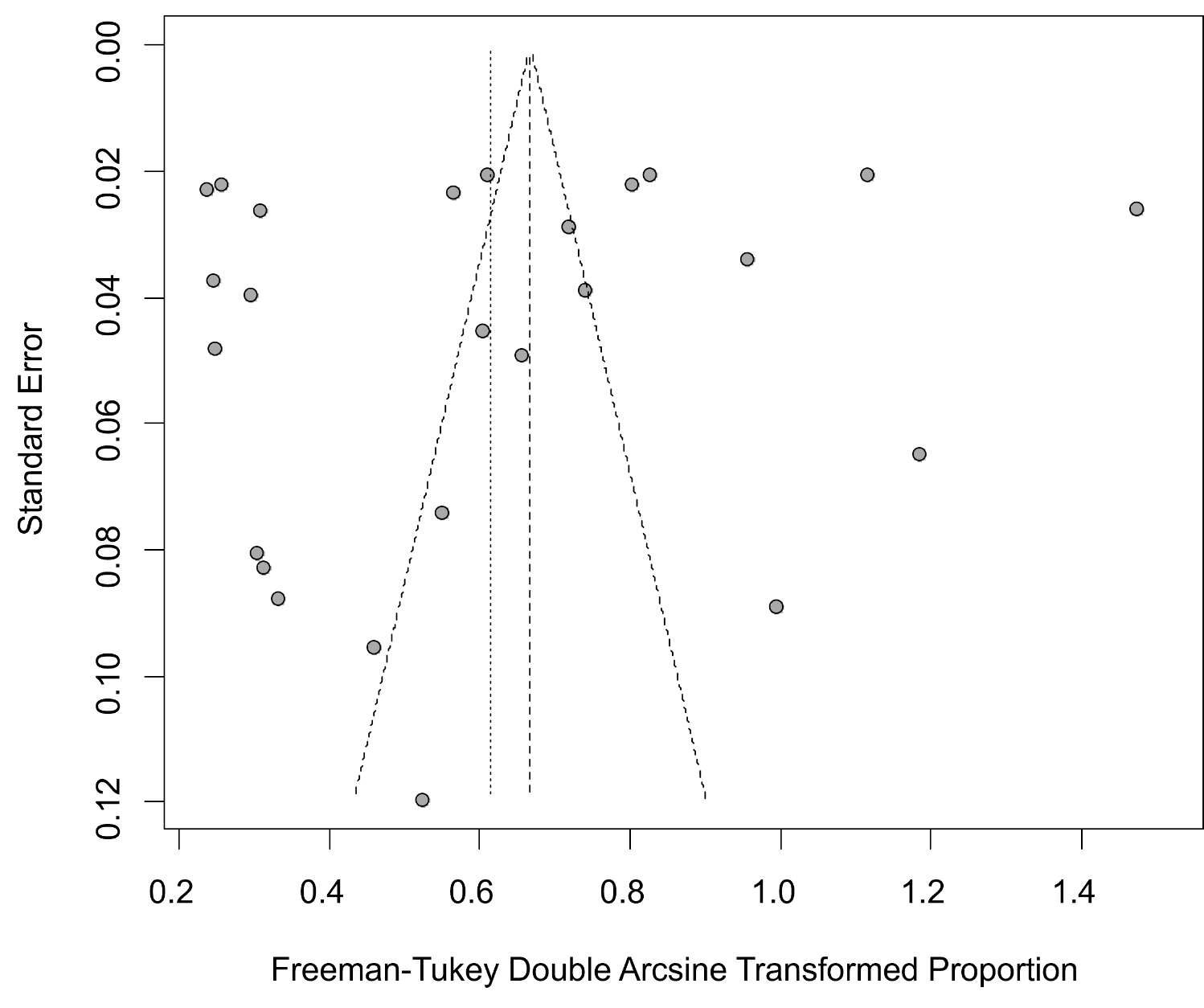


**Figure S7. Funnel plot with pseudo 95% confidence limits intervals for the examination of publication bias of gender**


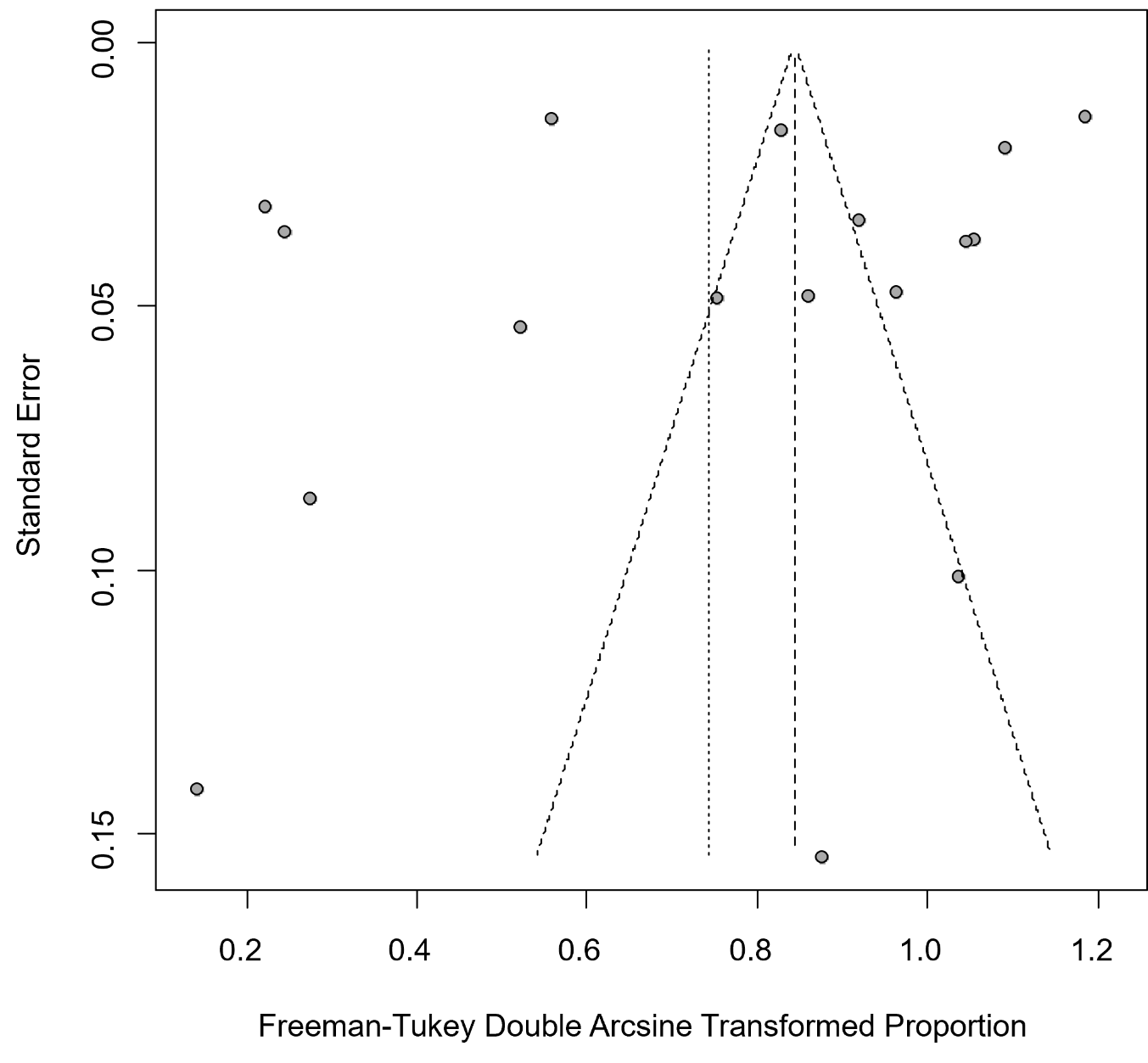


**Figure S8. Funnel plot with pseudo 95% confidence limits intervals for the examination of publication bias of age**


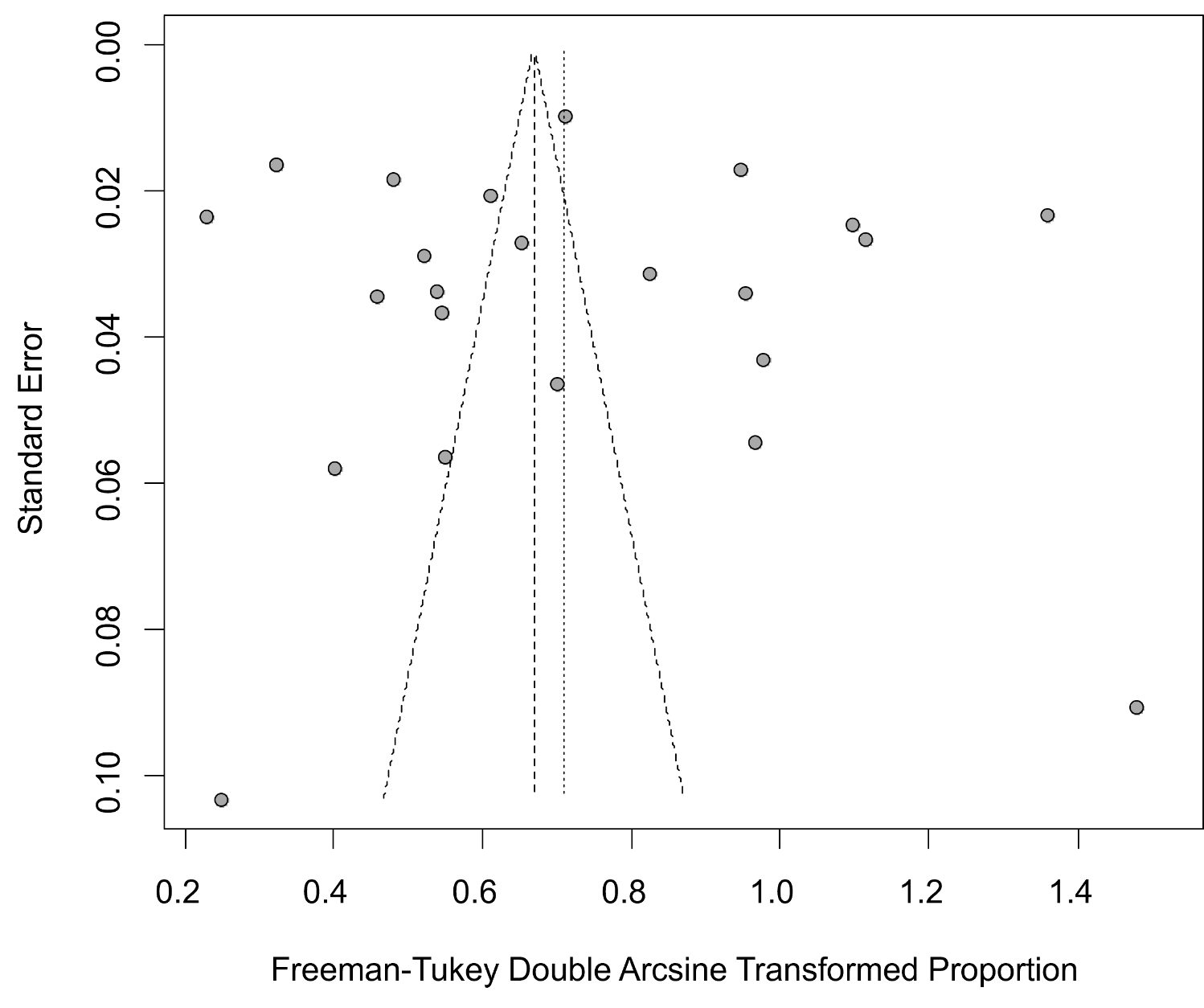


**Figure S9. Funnel plot with pseudo 95% confidence limits intervals for the examination of publication bias of study quality**


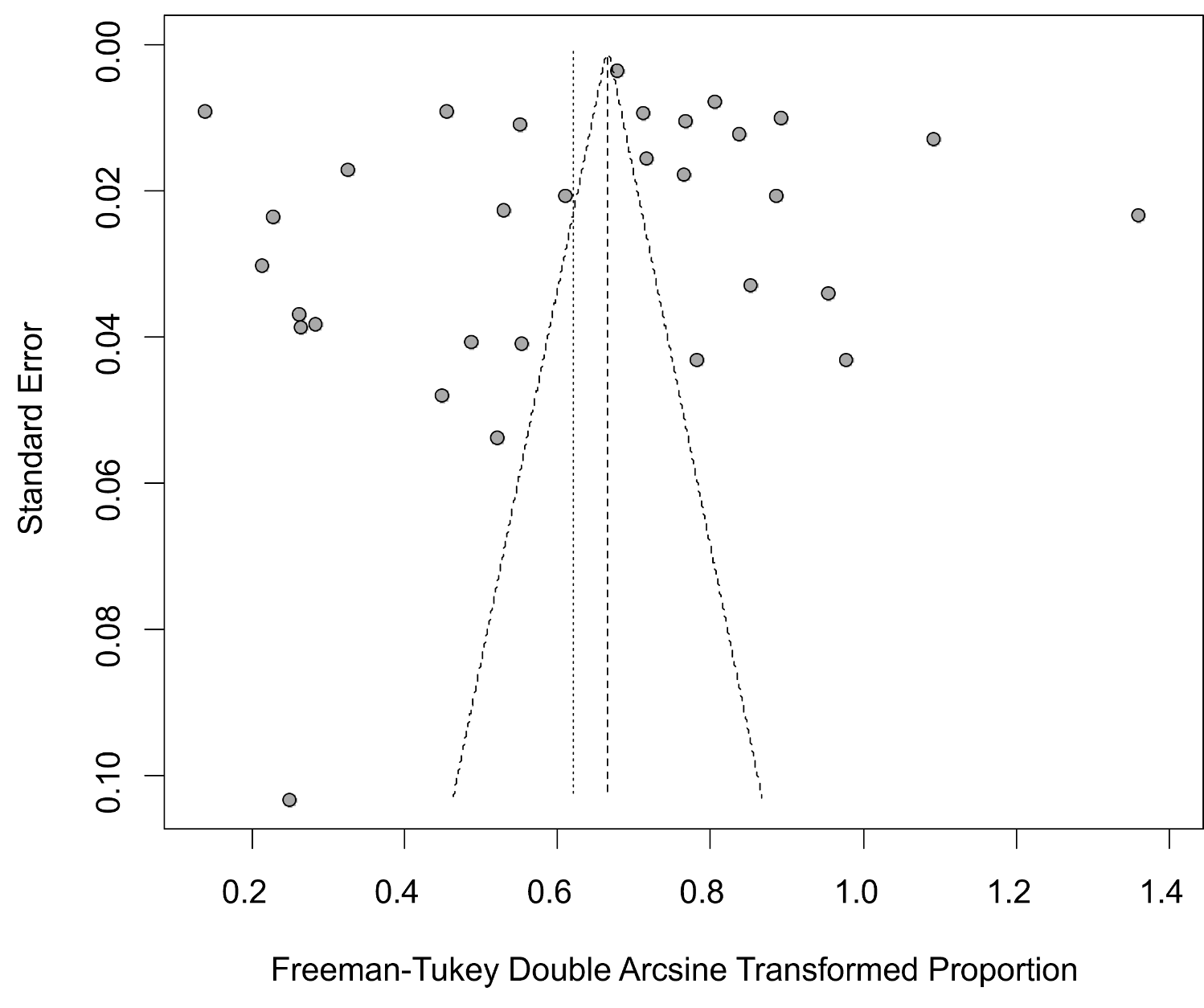


**Figure S10. Funnel plot with pseudo 95% confidence limits intervals for the examination of publication bias of province**


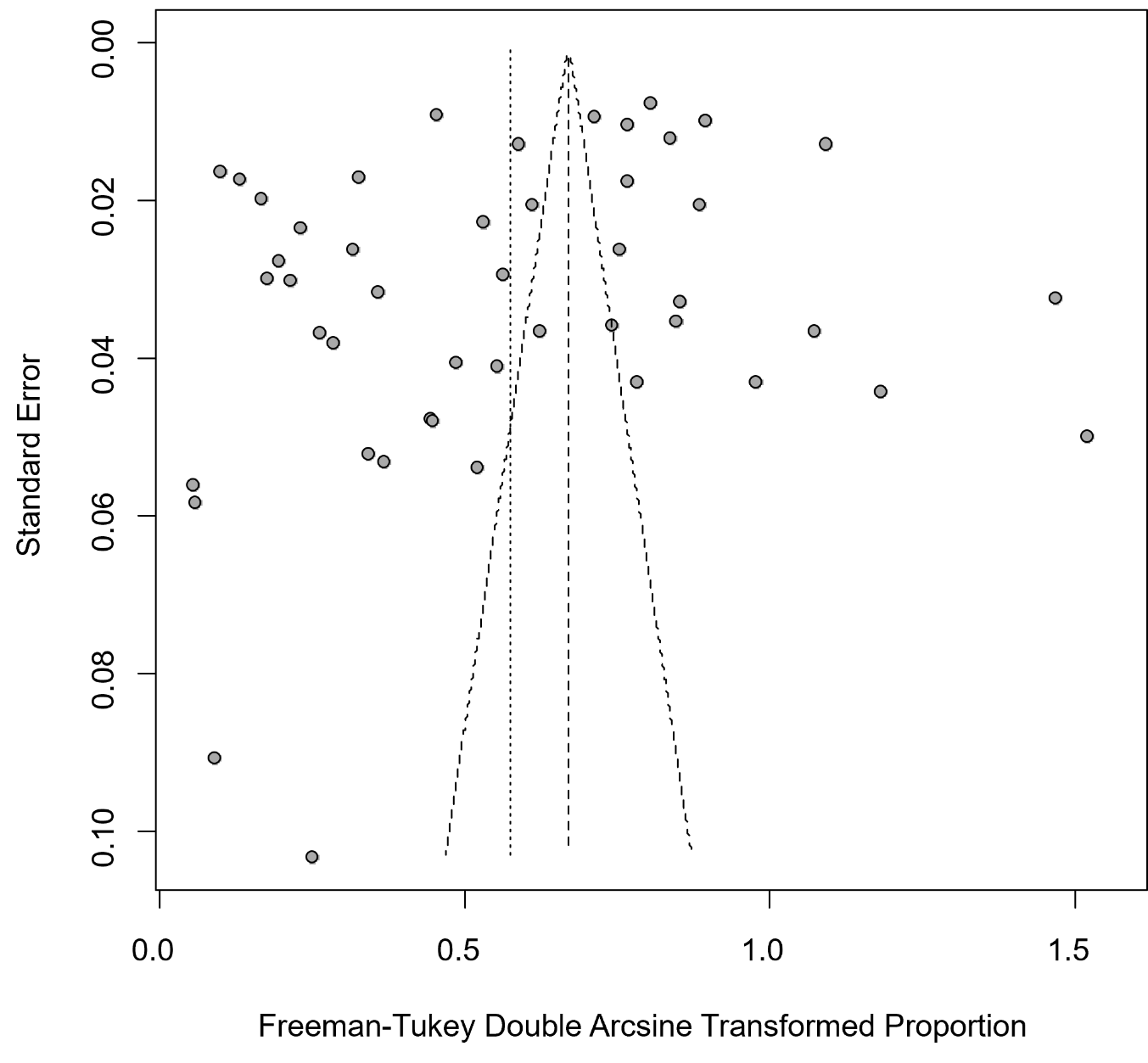


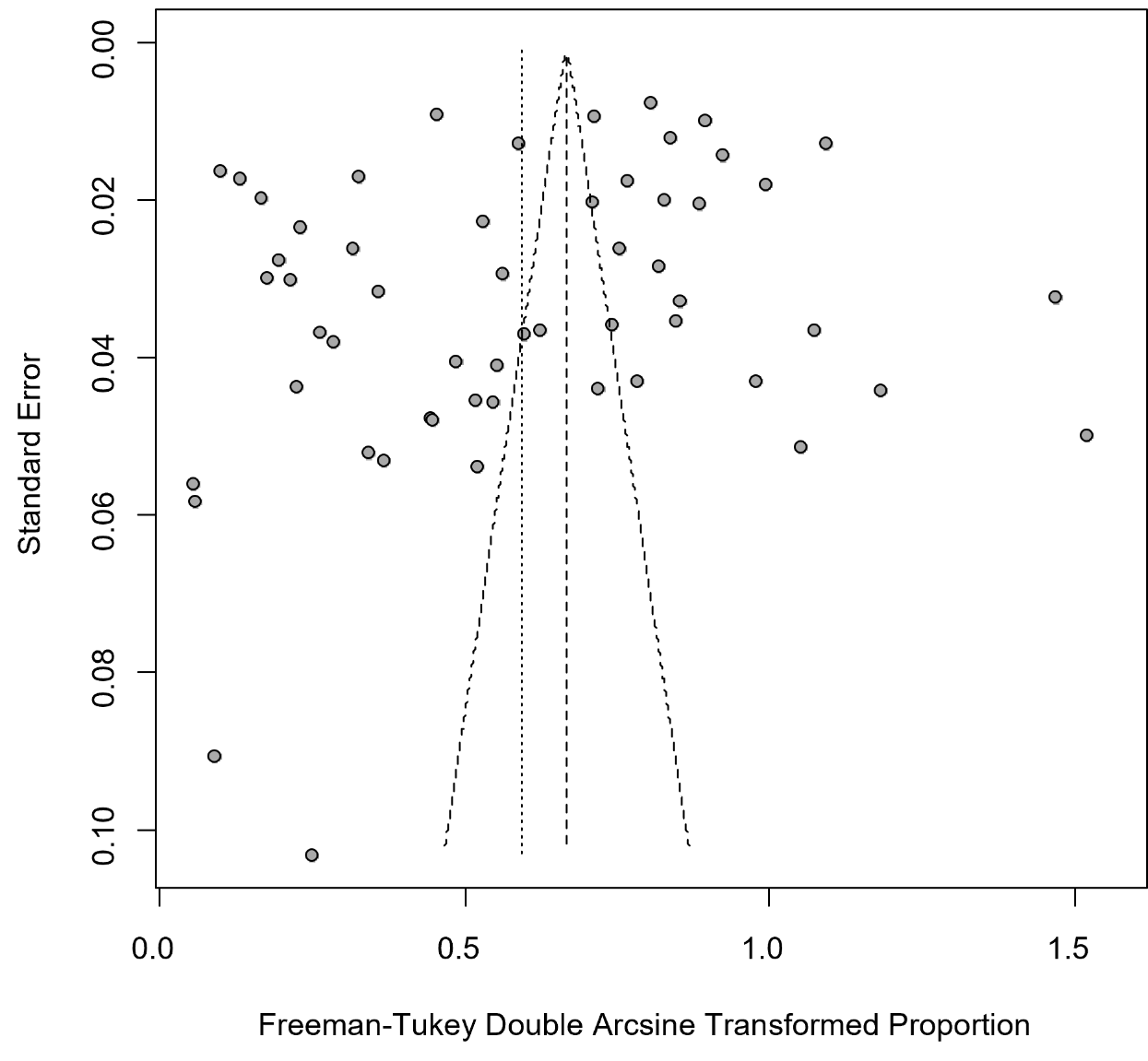
**Figure S11. Funnel plot with pseudo 95% confidence limits intervals for the examination of publication bias of latitude**


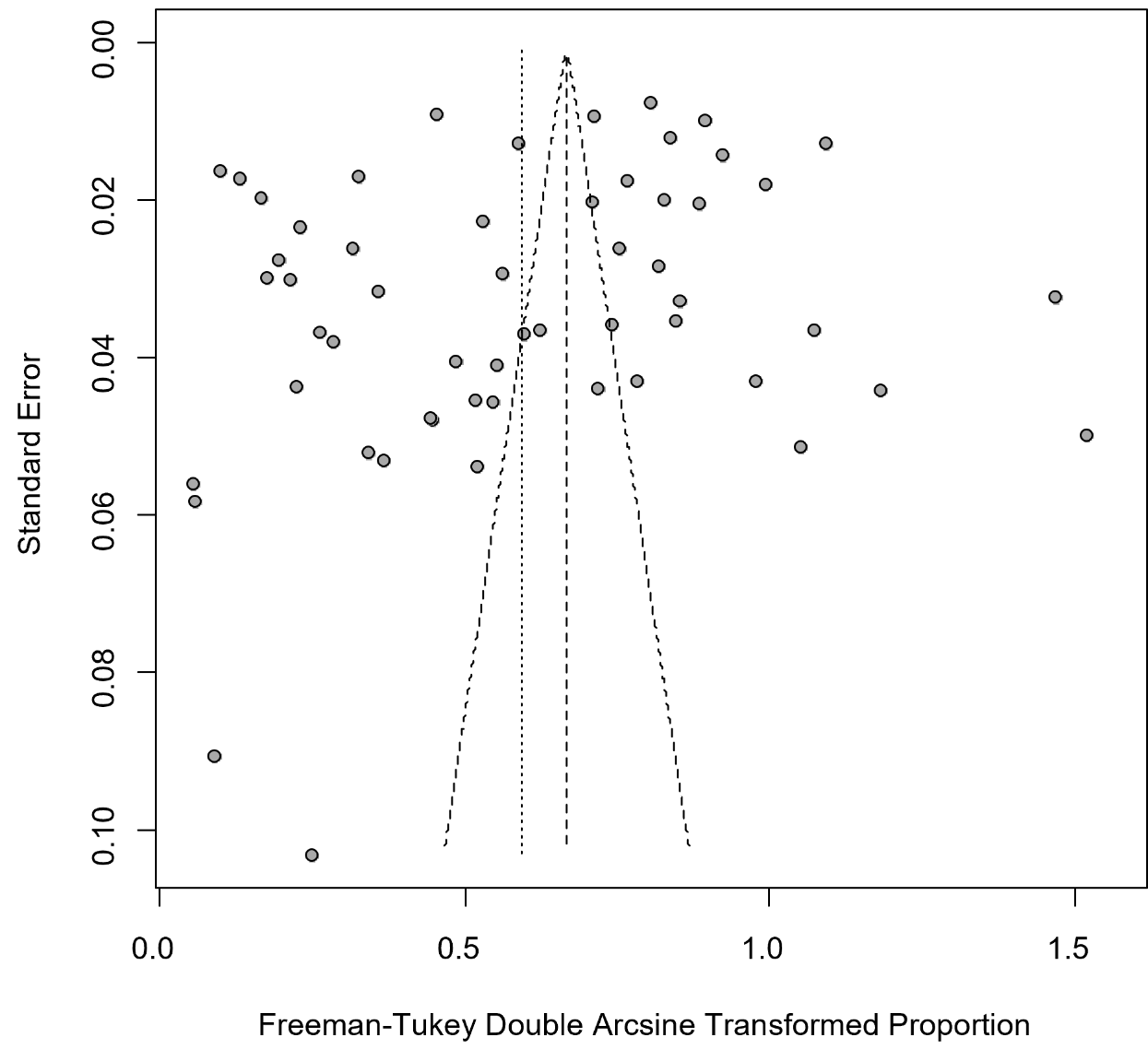
**Figure S12. Funnel plot with pseudo 95% confidence limits intervals for the examination of publication bias of longitude**

**Figure S13. Funnel plot with pseudo 95% confidence limits intervals for the examination of publication bias of altitude**


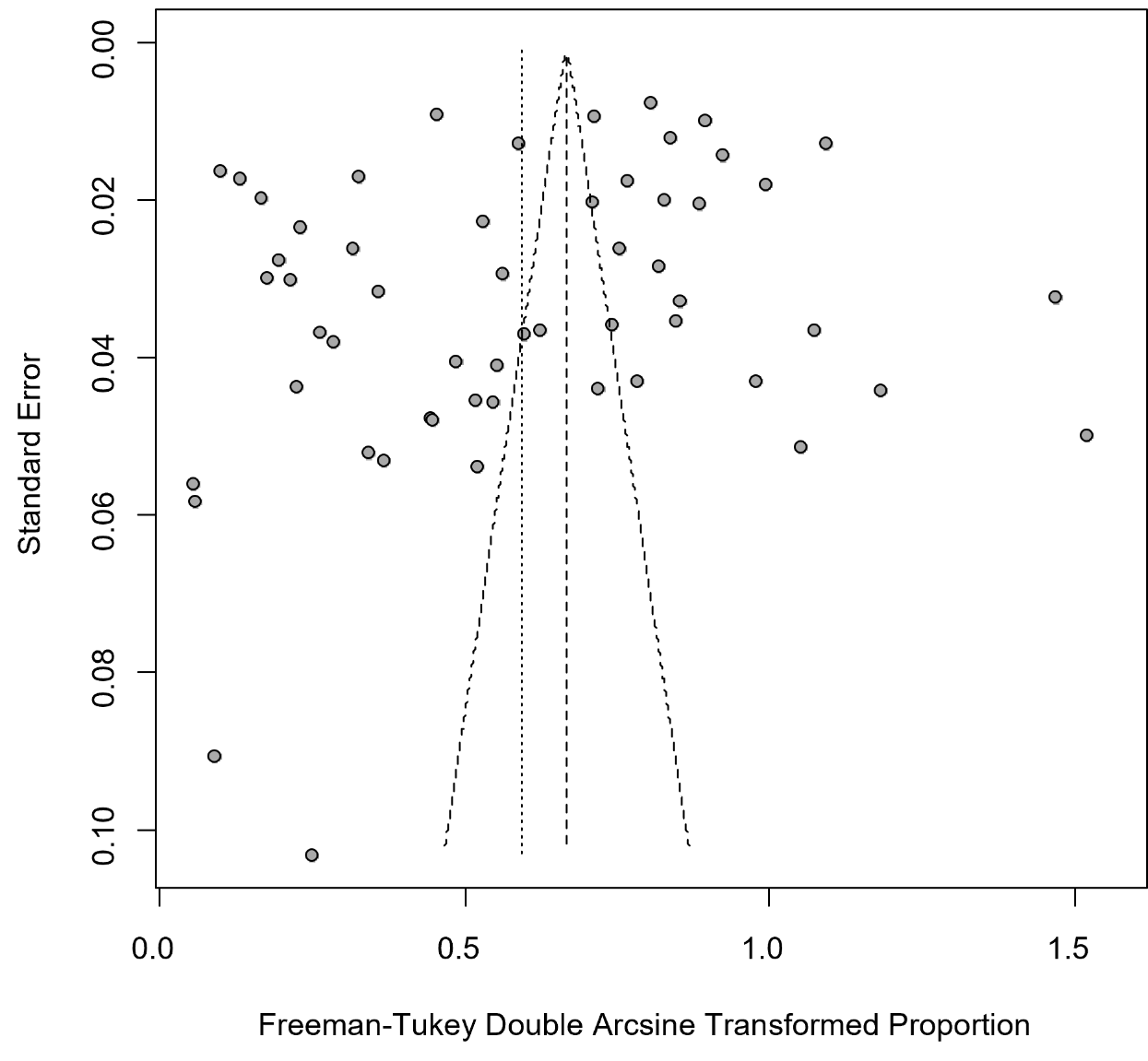


**Figure S14. Funnel plot with pseudo 95% confidence limits intervals for the examination of publication bias of rainfall**


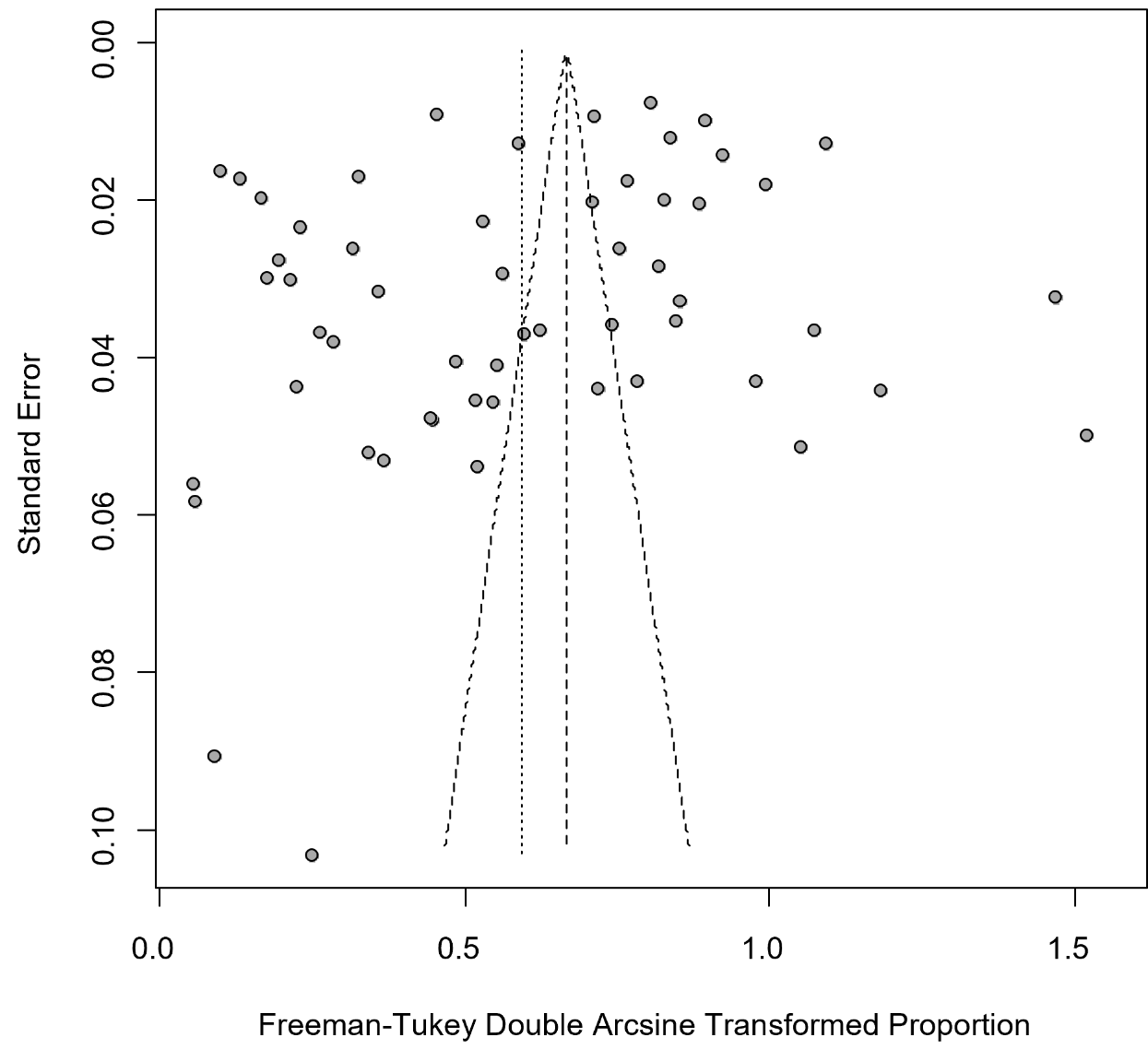


**Figure S15. Funnel plot with pseudo 95% confidence limits intervals for the examination of publication bias of humidity**


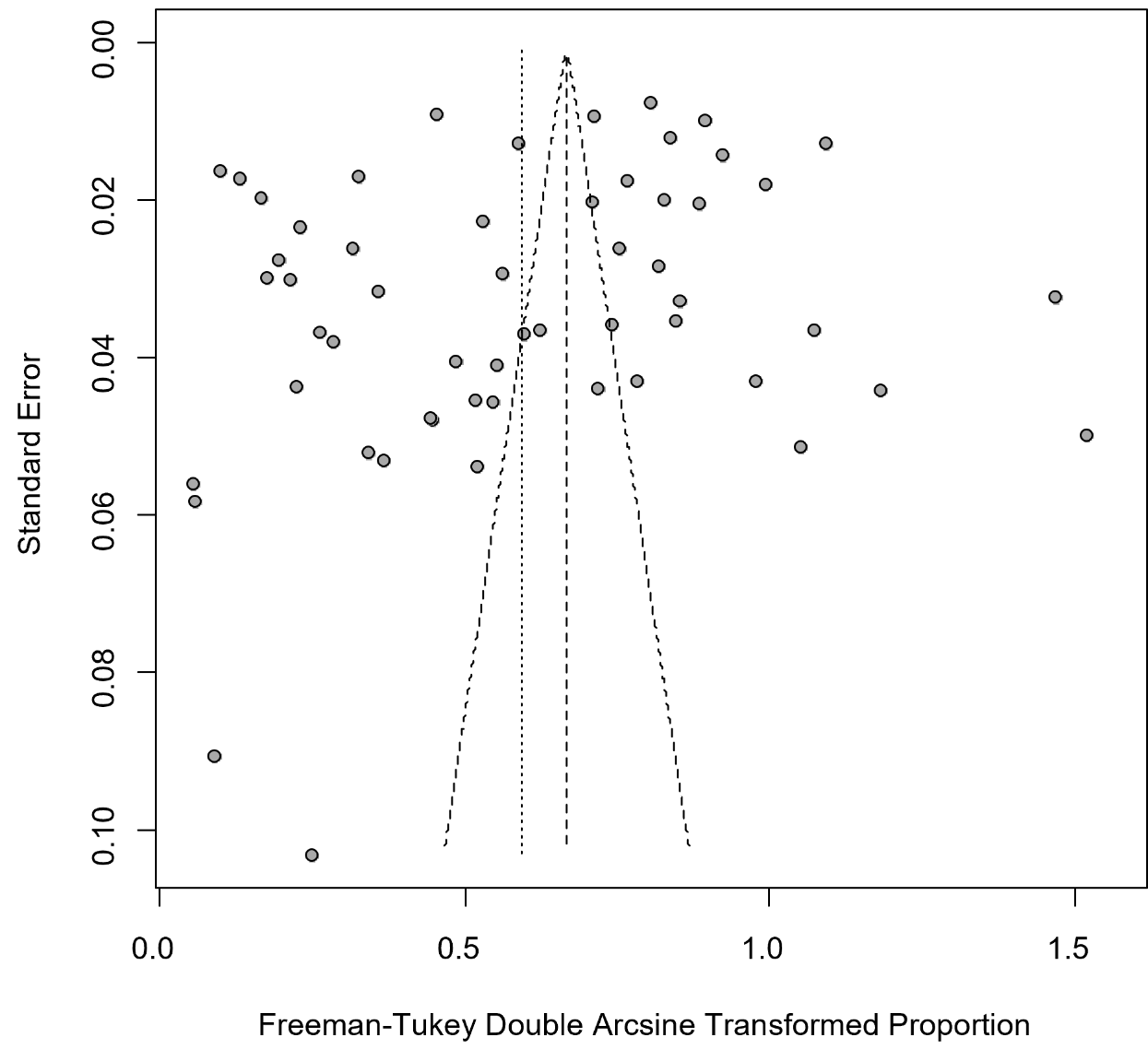


**Figure S16. Funnel plot with pseudo 95% confidence limits intervals for the examination of publication bias of minimum annual temperature**


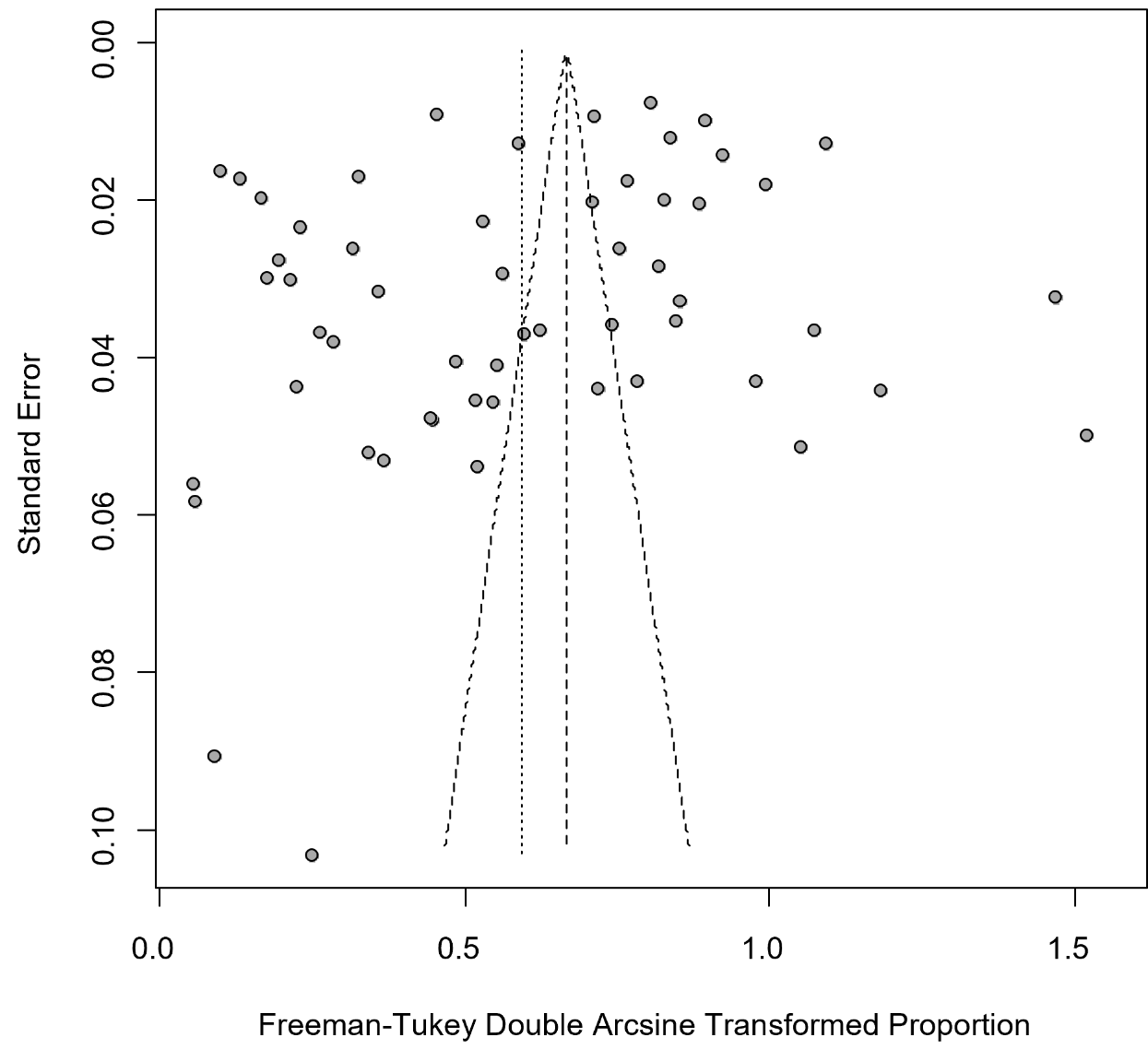


**Figure S17. Funnel plot with pseudo 95% confidence limits intervals for the examination of publication bias of maximum annual temperature**


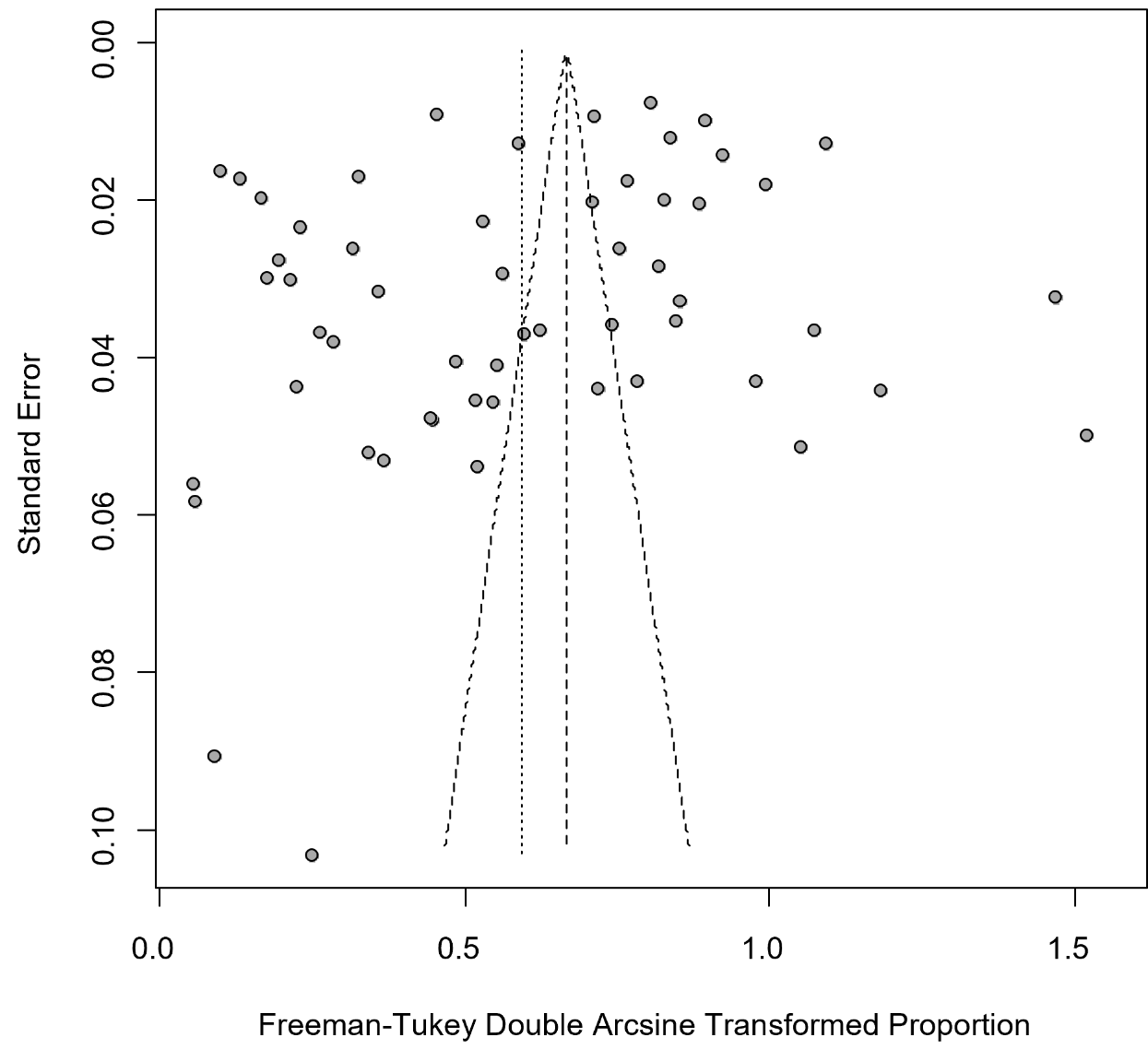


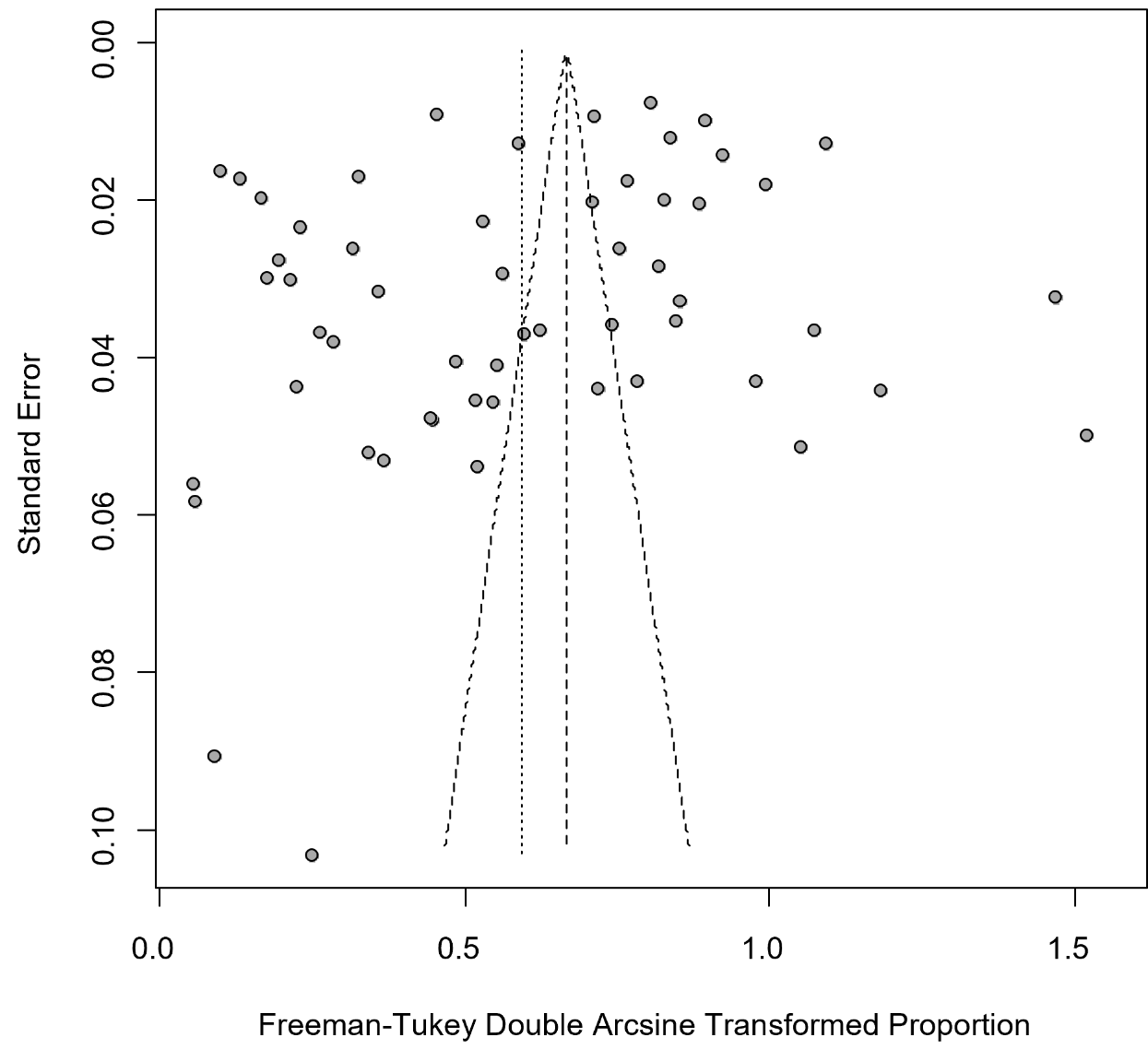
**Figure S18. Funnel plot with pseudo 95% confidence limits intervals for the examination of publication bias of average annual temperature**

**Figure S19. Funnel plot with pseudo 95% confidence limits intervals for the examination of publication bias of climate**


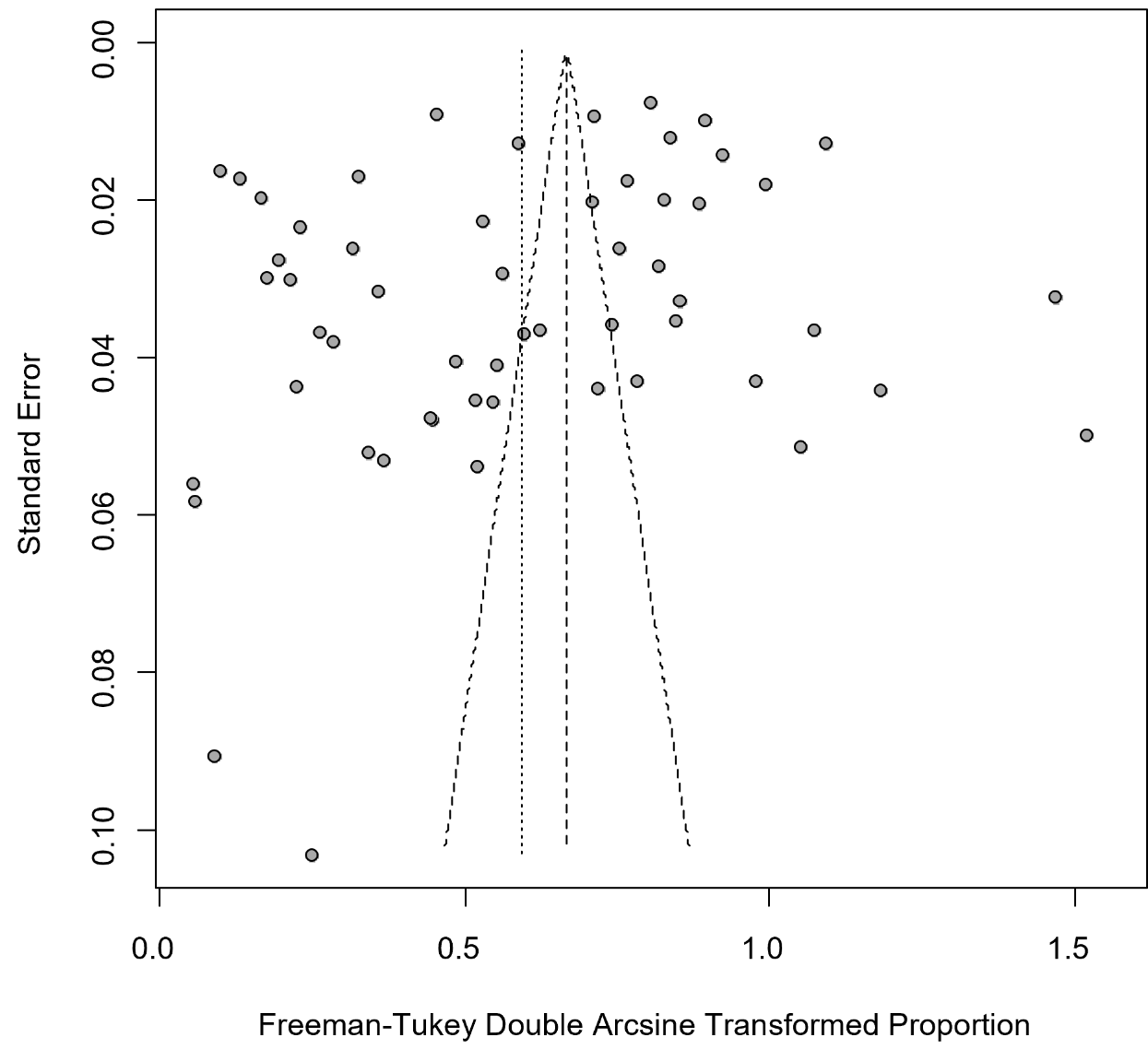

Supplement: Supplementary file 1 [file Supplementary_file_1.docx]
